# Haplotype-resolved genome of *Mimosa bimucronata* revealed insights into leaf movement and nitrogen fixation

**DOI:** 10.1186/s12864-024-10264-8

**Published:** 2024-04-03

**Authors:** Haifeng Jia, Jishan Lin, Zhicong Lin, Yibin Wang, Liangwei Xu, Wenjie Ding, Ray Ming

**Affiliations:** 1 College of Agriculture, Center for Genomics and Biotechnology, Fujian Agriculture and Forestry University, Fuzhou, 350002 China; 2 National Key Laboratory for Tropical Crop Breeding, Institute of Tropical Bioscience and Biotechnology, Chinese Academy of Tropical Agricultural Sciences, Haikou, 570100 China; 3 College of Environment and Biological Engineering, Putian University, Putian, 351100 China; 4 Agricultural Genomics Institute at Shenzhen, Chinese Academy of Agricultural Sciences, Shenzhen, 518120 China; 5 College of Life Science, Fujian Agriculture and Forestry University, Fuzhou, 350002 China

**Keywords:** *Mimosa bimucronata*, Genome assembly, Leaf movements, Nitrogen fixation

## Abstract

**Background:**

*Mimosa bimucronata* originates from tropical America and exhibits distinctive leaf movement characterized by a relative slow speed. Additionally, this species possesses the ability to fix nitrogen. Despite these intriguing traits, comprehensive studies have been hindered by the lack of genomic resources for *M. bimucronata*.

**Results:**

To unravel the intricacies of leaf movement and nitrogen fixation, we successfully assembled a high-quality, haplotype-resolved, reference genome at the chromosome level, spanning 648 Mb and anchored in 13 pseudochromosomes. A total of 32,146 protein-coding genes were annotated. In particular, haplotype A was annotated with 31,035 protein-coding genes, and haplotype B with 31,440 protein-coding genes. Structural variations (SVs) and allele specific expression (ASE) analyses uncovered the potential role of structural variants in leaf movement and nitrogen fixation in *M. bimucronata*. Two whole-genome duplication (WGD) events were detected, that occurred ~ 2.9 and ~ 73.5 million years ago. Transcriptome and co-expression network analyses revealed the involvement of aquaporins (AQPs) and Ca^2+^-related ion channel genes in leaf movement. Moreover, we also identified nodulation-related genes and analyzed the structure and evolution of the key gene NIN in the process of symbiotic nitrogen fixation (SNF).

**Conclusion:**

The detailed comparative genomic and transcriptomic analyses provided insights into the mechanisms governing leaf movement and nitrogen fixation in *M. bimucronata*. This research yielded genomic resources and provided an important reference for functional genomic studies of *M. bimucronata* and other legume species.

**Supplementary Information:**

The online version contains supplementary material available at 10.1186/s12864-024-10264-8.

## Background

The genus *Mimosa* is one of the most diverse genera in the Fabaceae family with more than 530 species, which is predominant in the Neotropics with some species distributed in Madagascar and a few in East Africa and South Asia [1, 2]. The origin of the genus was in Central and South America and most species are distributed in the tropics at low and medium altitudes, with some species also reaching subtropical or warm temperate regions [3, 4]. There are two centers of diversity for this genus, one located in the central region of Brazil, particularly in the Cerrado region with high species diversity, and the other in the central and southern regions of Mexico [5, 6]. Many species of this genus are economically important and are used for buildings and carpentry, for cellulose and fuel [7], as well as for soil enrichment and restoration of degraded land due to their nitrogen-fixing capacity [8]. Species in *Mimosa* have a wide range of adaptations to different environments, resulting in different growth forms and light requirements, which in turn leads to a great diversity of species [2]. Therefore, the genus *Mimosa* has long been used as a study system for biogeographical and macroevolutionary studies. It is particularly known for having evolved frequent adaptations to fire to survive in fire-prone environments such as the Brazilian Cerrado [9], and for frequently alternating between humid and dry tropical biomes [10]. *Mimosa* is best known for its seismonastic leaf movement and the hypothesis that seismonasty occurs independently in eight lineages of *Mimosa* [2]*.* For instance, *Mimosa pudica* belongs to clade P and shows rapid leaf movement in response to touch within seconds. However, the trait is also present in many other *Mimosa* species depending on a lesser degree. *Mimosa bimucronata*, for example, which belongs to clade D, shows slow leaf movement in response to touch within minutes [2]. The biological significance of seismic leaf movement is still not fully understood. A recent study demonstrates that it indeed can be a defense mechanism against herbivores [11], but whether or not it is a protective mechanism to protect leaves from damage in regions with humid climates needs further investigation. A study found that species in *Mimosa* can be considered precursors to earthquakes [12].

*M. bimucronata* has become an aggressive invasive species after its introduction to China [13]. Two other species*,* including *M. pudica and M. pigia*, are the most widespread *Mimosa* species, and *M. pigia* is one of the worst invasive species in the world due to its numerous characteristics that favour invasion [14]. *M. pudica* is the most widespread species, characterized by rapid leaf movement, and *M. bimucronata* is known for its usefulness as a revetment and embankment plant with characteristics of rapid growth and resistance to waterlogging. *M. bimucronata* belongs to the subfamily Caesalpinioideae of the legume family [15]. *M. bimucronata* has a reputation as the "king of hedgerows", known for its strong adaptability to the environment and for the characteristics of strong stress resistance and fast growth. *M. bimucronata* also has some ornamental value with dense spherical inflorescences and white, fragrant flowers. Compared to *M. pudica*, the leaves of *M. bimucronata* are less sensitive to external stimuli, which is advantageous for research into the molecular mechanism of leaf movement, as sufficient time was available to collect samples before and after touch. Additionally, *M. bimucronata* and *M. pudica*, which are in a relatively distant clade (clade D and F, respectively) [2], showed the trait of leaf movement in response to external stimuli at different rates, suggesting that the trait has a different genomic basis in the two species. It is necessary to obtain more information about the *M. bimucronata* genome to analyze the trait from an evolutionary perspective. Chromosome number is an important trait for species classification and genome composition. Previous studies have indicated that x = 13 is the basic chromosome number in the genus *Mimosa*, which forms the basis for ploidy determination and provides guidance for anchoring the assembled genomes [16].

The movement of plants is an interesting phenomenon and is generally slow. However, some plant species have evolved the ability to exhibit rapid movements comparable to those of animals, such as *Dionaea muscipula* and *M. pudica* [17]*.* Turgor pressure is the primary driving force, and elastic forces are secondary for many plant movements. Generally, ion channels, Ca^2+^, and water in and out of the cell are the main factors affecting turgor pressure, involved in plant movement [18, 19]. Both aquaporins (AQPs) located in the plasma membrane and tonoplast contribute to seismonastic leaf movements in *M. pudica* [20]. Calmodulin-like (CML) and xyloglucan endotransglucosylase/hydrolase (XTH) genes respond to touch and dark stimuli, and these stimuli have partially overlapping signal transduction pathways [21]. Mechanical and chemical stimuli associated with predators can induce dynamic changes in Ca^2+^ signaling in carnivorous sundew leaves [22]. Ca^2+^ sensors transmit Ca^2+^ signals downstream, triggering a cascade response that regulates plant growth and development and its response to the environment [23]. Rapid deformation resulting from turgor pressure is only one of the steps leading to the movement of plant leaves. Systems for rapid detection and transmission of signals are also needed. In a way, these studies provided chemical and molecular bases for leaf movements. However, how often this trait of seismonic leaf movements has evolved in *Mimosa* remains unknown, and genomics is an important and effective way to study plant evolution.

Throughout evolution, rhizobia and legumes have established a unique mutualistic symbiosis characterized by symbiotic nitrogen fixation (SNF) and the most recent common ancestor can be dated to 90 million years ago [24]. SNF is another important phenomenon in most species of the Fabaceae family, involving complex mechanisms and interactions [25, 26]. There are nearly 200 genes required for SNF, that have been discovered by various forward and reverse genetic approaches [27]. Genes associated with SNF in plants have been categorized into two groups, including symbiotic genes (Sym genes) and nodulin genes [28]. Sym genes are mainly involved in the processes of response to bacterial signals, symbiosis signal transduction, and nodule infection, while nodulin genes are mainly involved in the processes of nodule organogenesis, development, and nitrogen fixation [24, 27]. Over 50 Sym genes have been identified in model legumes such as *Medicago truncatula* and *Lotus japonicus* [27]. The first common symbiotic gene, namely, *LjSYMRK*/*MtDMI2*, is essential for the process of rhizobial and mycorrhizal symbioses [29]. Two other well-studied symbiotic genes are Nod factor receptor 1 (*LjNFR1*/*MtLYK3*) and Nod factor receptor 5 (*LjNFR5/MtNFP*), which co-operate with *LjSYMRK*/*MtDMI2* to initiate nodule organogenesis and bacterial infection [27, 30]*.* Moreover, *LjNFR5* can activate the Nodule Inception (NIN) gene through phosphorylation [31]. The key nodulation genes, namely, *LjNIN*, *MtRPG*, and *LjNFR1/MtLYK3* have been lost in the non-nodulating species, suggesting that these three genes play a crucial role in the process of root nodulation for nitrogen fixation and provide a reference for other legume in nitrogen fixation by root nodules [25, 32]. A recent study shows that the genes *LjNFR5*/*MtNFP* and *NIN* are pseudogenised in non-nodulating species, indicating that these two genes have lost their original function and form a new function in nodulating nitrogen-fixing species [33]. However, nitrogen fixation in the nodules of *M. bimucronata* has not been systematically studied*,* especially for the key gene NIN, which is involved in the processes of symbiosis and nitrogen fixation. The objectives of this project are to sequence the genome of *M. bimucronata* and to uncover the genomic basis for leaf movement and nitrogen fixation in this species.

## Results

### Genome sequencing, assembly and annotation

**Fig. 1:**
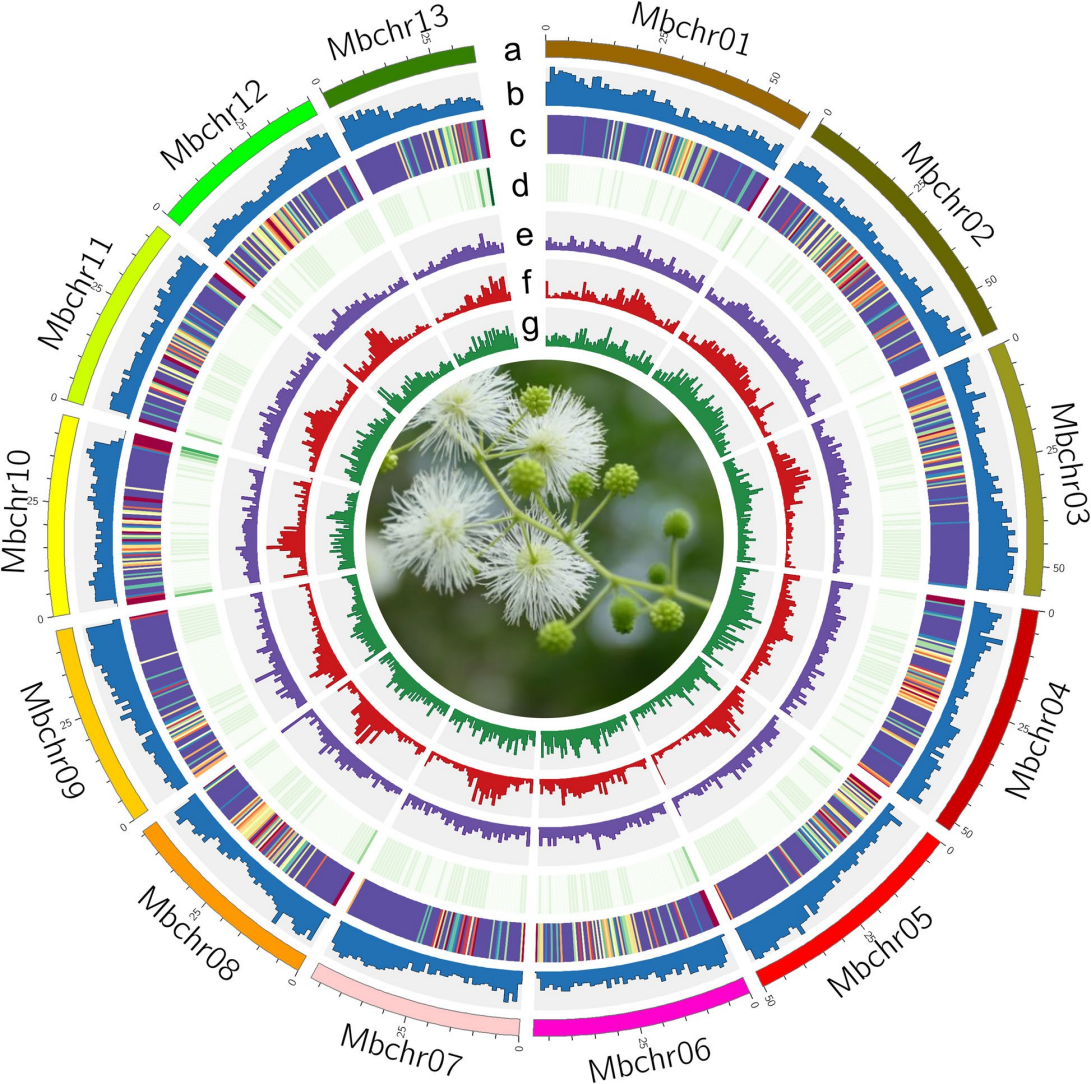
The landscape of genome features of *M. bimucronata.* Outer to inner tracks: (**a**) 13 chromosomes (Mbchr01-Mbchr13), (**b**) Gene density, (**c**) Gene expression, purple indicates high expression level and yellow means low. The expression data were from six samples, including root, SAM, stem, and three states of leaf, each with three biological replicates. **d** GC contents, (**e**) DNA transposable element abundance, (**f**) LTR/Copia abundance, (**g**) LTR/Gypsy abundance. All distributions are drawn in a window size of 1 Mb

*M. bimucronata* is diploid with x = 13 (Supplementary Fig. 1), and its genome size is approximately 660 Mb as estimated by flow cytometry (FCM) (Supplementary Table 1, Supplementary Fig. 2) and approximately 654.60 Mb by K-mer analysis based on Illumina data (Supplementary Table 2, 3 and 4, Supplementary Fig. 3). Subsequently, a PacBio HiFi library was constructed and sequenced, generating 17.34 Gb long read sequences with 26 × coverage (Supplementary Table 4). Additionally, a Hi-C library was constructed and sequenced, resulting in 59.27 Gb of clean data (Supplementary Table 5 and 6). The assembled genome was approximately 648 Mb in size with a contig N50 at 19 Mb and a scaffold N50 at 48 Mb. This assembly served as the reference genome for the subsequent analyses (Table 1 and Supplementary Table 7). A chromosome-level assembly was generated with 13 pseudochromosomes anchoring 627 Mb (96.66%) of the genome (Fig. 1, Supplementary Fig. 4, Supplementary Table 8). The haplotype-resolved assemblies at the chromosome level resulted in haplotype A and haplotype B, each with a contig N50 of 6.57 Mb and 6.88 Mb, respectively (Table 1).

**Table 1:** Summary of genome assembly, annotation, and assessments of *M. bimucronata*

Descriptions	Monoploid genome assembly	Haplotype-resolved chromosomal-level	
		**Haplotype A**	**Haplotype B**
Estimated genome size (Mb)	660	/	/
Assembly size (Mb)	648.36	599.96	619.79
Percent of estimated genome size (%)	98.2	91	93.9
Contig N50 (Mb)	19.04	6.57	6.88
Scaffold N50 (Mb)	48.41	44.4	48.5
BUSCO completeness of assembly (%)	98.7	97.4	98.4
Total number of genes	32,146	31,035	31,440
BUSCO completeness of annotation (%)	98.4	95.8	97.4
Raw LAI	11.87	11.4	11.52
LAI	15.27	13.54	13.4
QV	43.9	44.62	44.99
K-mer completeness	86.82	84.83	86.88
Phase blocks and switch error rate	4.07E-05	3.45E-05	3.17E-05
False duplications	2.58	2.49	2.96
Number of genes with two alleles	/	14,685	
Number of genes with the same allele	/	9,929	
Number of genes with one allele	/	1,653	2,061
Total number of anchored genes	31,824	30,164	30,589
Unanchored genes or alleles	322	871	851
Sequence identities (median)	/	99.40%	
Sequence identities (average)	/	99.40%

Illumina sequences (~ 41 ×) were mapped to the assembled genome, resulting in a mapping rate of 96.47% and a coverage rate of 99.99% (Supplementary Table 9). Transcriptome sequences were mapped to the genome to evaluate sequence integrity, achieving an accuracy of 99.99% (Supplementary Table 10). The completeness of the genome was assessed by Benchmarking Universal Single-Copy Orthologs (BUSCO, v3.0.2). A total of 98.70% (1593 out of 1614 BUSCOs) of the plant-specific orthologs were identified as complete, and 94.40% (1,523 out of 1,614 BUSCOs) were single-copy BUSCOs (Table 1, Supplementary Table 11). The genome exhibited a Long Terminal Repeat Retrotransposons (LTR) Assembly Index (LAI) value of 15.27 (10 ≤ LAI < 20) (Table 1), reaching the quality of a reference genome. BUSCO analysis of the genomes of haplotype A and haplotype B yielded 97.4% and 98.4%, respectively (Table 1, Supplementary Table 12). The LAI values for haplotypes A and B were 13.54 and 13.40, respectively (Table 1). Both haplotype genomes exhibited consensus quality values (QV) greater than 40 (Table 1).

Homology-based approaches, de novo approaches, and transcriptome-based approaches were integrated for the prediction of protein-coding genes, and 32,146 genes were annotated. Among them, 31,137 (96.86%) genes were functionally annotated against different functional databases (Supplementary Table 13). The BUSCO completeness of the annotation was 98.40% (Supplementary Table 11). We further annotated non-coding RNA genes, yielding 165 tRNA, 99 miRNA, 39 rRNA, and 336 snRNA of the monoploid genome (Supplementary Table 14). For the haplotype A and B genomes, 31,035 and 31,440 genes were annotated, respectively, with the BUSCO completeness of annotation being 95.8% and 97.4%, respectively (Table 1).

The repetitive sequences were comprehensively characterized, revealing that 57.04% of the assembled monoploid genome of *M. bimucronata* consisted of repetitive elements. Notably, Long Terminal Repeat Retrotransposons (LTRs) were the most abundant, accounting for 22.99% of the genome assembly (Supplementary Table 15). The repetitive sequences accounted for 58.27% and 58.57% of the haplotype A and B genomes, respectively (Supplementary Table 16).

### Genome evolution and phylogenetic analysis

**Fig. 2:**
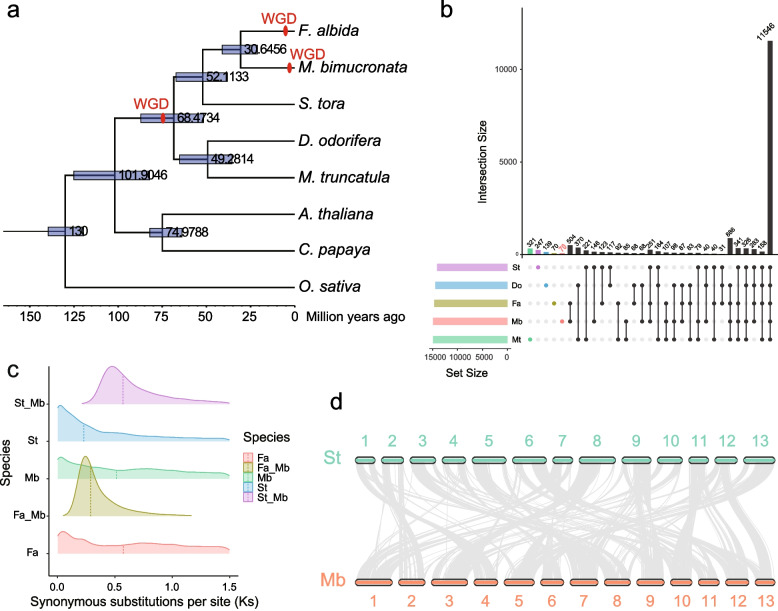
Phylogenetic and gene family analysis of *M. bimucronata* and other representative plant genomes. **a** Estimation of the divergence time between 8 species. The length of the blue bar represents the range of the divergence time. The numbers at internodes show the median divergence time (million years ago, Mya) with a 95% confidence interval [CI]. One recent WGD event occurred at 6.8 Mya in *M. bimucronata* and *F. albida* is indicated by the red color. **b** Upset diagram showing the distribution of shared gene families among *M. bimucronata* and the other four species. Gene family complements comparisons among *M. bimucronata*, *F. albida*, *S. tora*, *D. odorifera*, and *M. truncatula*. There were 11,546 common gene families and 70 M*. bimucronata*-specific gene families. **c** WGD analysis of the *F. albida*, *S. tora*, and *M. bimucronata* genomes. The distribution of synonymous substitution rates (Ks) was analyzed for paralogs within *M. bimucronata*, *S. tora,* and *F. albida,* as well as orthologs between *M. bimucronata* and the other two genomes. Peaks in the Ks distribution of orthologs imply the potential time of species divergence, while a peak for paralogs indicates a whole genome duplication (WGD) event. **d** *S. tora* and *M. bimucronata* synteny showing the span of their shared regions. The gray line connects matched gene pairs

The genome assembly of *M. bimucronata* was compared with the genomes of seven other sequenced plant genomes, including four genomes from the legume family (*Faidherbia albida*, *Senna tora*, *Dalbergia odorifera*, and *Medicago truncatula*), as well as genomes of *Arabidopsis thaliana*, *Carica papaya* and *Oryza sativa* (Supplementary Table 17)*.* These seven species were selected for the reason that four species are related species of *M. bimucronata* and the other three species were used for calibration. A total of 920 single-copy orthologs were identified and used to construct the species phylogenetic tree (Fig. 2a, Supplementary Table 18). *M. bimucronata* was relatively close to *F. albida* and *S. tora*, and the divergence times were 30.6 million years ago (mya) and 52.1 mya, respectively (Fig. 2a), which is mainly because all these three species belong to the subfamily Caesalpinioideae*,* whereas the other two legume species belong to the subfamily Papilionoideae. Among the five species of the legume family, 11,546 gene families were shared, and 252 genes in 70 gene families were specific to the *M. bimucronata* genome (Fig. 2b, Supplementary Table 19 and 20). These species-specific genes were distributed across all 13 chromosomes of *M. bimucronata* (Supplementary Fig. 5). Gene Ontology (GO) and Kyoto Encyclopedia of Genes and Genomes (KEGG) enrichment analyses were performed on these species-specific genes. The functions of these species-specific genes were mainly enriched in GO terms of biological processes, such as the plastoquinone biosynthetic process, negative regulation of endopeptidase activity, and negative regulation of peptidase activity. For the KEGG analysis, these species-specific genes were enriched in the nucleocytoplasmic transport pathway (Supplementary Fig. 6).

### Gene family expansion/contraction analysis

We examined gene family expansion and contraction in eight species included in the phylogenetic analysis. In the *M. bimucronata* genome, 1,817 gene families exhibited expansion, while 1,078 gene families showed contraction. Additionally, 222 gene families underwent rapid evolution (Supplementary Fig. 7 and Supplementary Table 21). GO and KEGG enrichment analyses were performed to further understand the functional implications of the rapidly evolving gene families. In the GO analysis, the rapidly evolving gene families were primarily associated with terpenoid metabolic processes and protein phosphorylation. In the KEGG enrichment analysis, the rapidly evolving gene families mainly evolved in the sesquiterpenoid and triterpenoid biosynthesis and glutathione biosynthesis pathways (Supplementary Fig. 8).

### WGD event analysis and collinearity

WGD events have major impacts on plant genome evolution [25, 34]. WGD was investigated in *M. bimucronata*, *F. albida* and *S. tora* (Fig. 2c). *M. bimucronata* and *F. albida* belong to the *Mimosoid* clade and share one ancient WGD event. The distribution of Ks values in *M. bimucronata* showed two peaks at Ks values of approximately 0.03 and 0.76. The first peak at 0.03 indicated that the most recent WGD event occurred ~ 2.9 million years ago (Mya), after the divergence of *M. bimucronata* and *F. albida,* later than the recent WGD of *F. albida*. The second peak which occurred at 73.5 Mya, was shared by *M. bimucronata* and *F. albida*, representing a much older WGD event.

The synteny between *M. bimucronata* and *S. tora* chromosomes appeared fragmented, revealing a 2:2 syntenic depth ratio (Supplementary Fig. 9). Specifically, chromosome 1 of *M. bimucronata* was found to be aligned with portions of chromosomes 7 and 10 of *S. tora*, whereas chromosome 2 of *M. bimucronata* was found to predominantly align with portions of chromosome 2 of *S. tora.* In general, the majority of the chromosomes of *M. bimucronata* were aligned with parts of the chromosome of *S. tora* in a one-to-one relationship (Fig. 2d).

### Structural variations between two haplotypes and allelic gene expression analysis

**Fig. 3:**
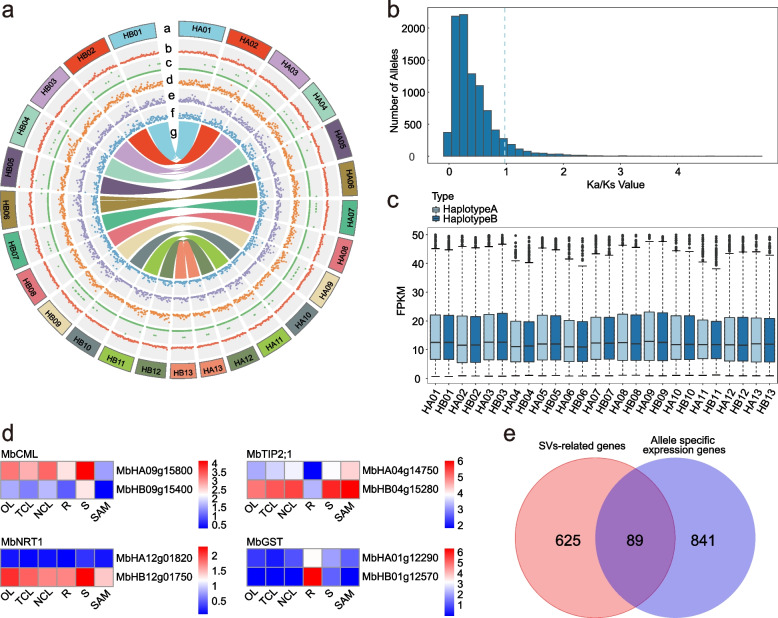
Structural variations and allelic gene comparisons between the two haplotypes. **a** Structural variations detected between two haplotypes and synteny analyses between allele genes in *M. bimucrnata.* Outer to inner tracks: (a) chromosome karyotype, (b) duplications (DUPs), (c) inversions (INVs), (d) inverted duplications (INVDPs), (e) inverted translocations (INVTRs), (f) translocations (TRANSs), (g) synteny analyses between allele genes. All distributions are drawn in a window size of 1 Mb. **b** Distribution of Ka/Ks between allele gene pairs. **c** Box plot of the FPKM (fragments per kilobase of transcript per million fragments mapped) value of allelic genes between two haplotypes. Light blue and dark blue represent haplotype A and haplotype B, respectively. **d** Four genes with different expression patterns in the two haplotypes. Red indicates high expression, and blue indicates low expression. The average FPKM of three biological replicates was used as the expression level, and they were normalized by Log2 (FPKM + 1). **e** Venn diagram of SV-related genes and allele-specific expression genes

A total of 7,606 structural variations (SVs) were detected between haplotypes A and B, including 3,202 duplications (DUPs), 20 inversions (INVs), 2,698 inverted duplications (INVDPs), 815 inverted translocations (INVTRs) and 841 translocations (TRANSs) (Fig. 3a and Supplementary Fig. 10). Among these SVs, INVs were specifically selected and manually validated using PacBio long reads and Illumina reads, confirming 20 inversions (Supplementary Fig. 11a). These SVs affected 714 genes in the *M. bimucronata* genome. GO analysis was performed with these SV-related genes (genes located in SV regions), and the genes were mainly enriched in the processes of regulation of proteolysis, regulation of hydrolase activity, response to herbivory, response to wounding and response to external biotic stimulus (Supplementary Fig. 12a). KEGG analysis results showed that SV-related genes were mainly enriched in the biology of nucleocytoplasmic transport, fatty acid biosynthesis, and phenylpropanoid biosynthesis (Supplementary Fig. 12b). Following the GO and KEGG analyses mentioned above, we proceeded to annotate the SV-related genes that exhibited significant enrichment. This annotation was intended to enhance our comprehension of the biological significance underlying these SVs. We observed that some genes from different types of SVs were involved in leaf movement and root nodule formation. For instance, *MbHA12g09480* from INV belongs to the multi antimicrobial extrusion (MATE) family, which might be involved in plant–microbe interactions and play important roles in the process of nodule formation, while *MbHA04g15050* from INVDP, a member of the Tre-2/Bub2/Cdc16 (TBC) domain family, could regulate leaf movement by regulating the small GTPases that are involved in the process of vesicle trafficking and membrane dynamics.

3.9 million SNPs and 222,701 small indels (< 50 bp), including 112,097 insertions and 110,604 deletions were detected (Supplementary Table 22), and these variations were widespread and randomly distributed across 13 pseudochromosomes (Supplementary Fig. 13). A total of 8,677 large indels (> 50 bp) were also detected, and 100 large indels on chromosome 1 were randomly selected for validation using PacBio long reads. Of these, 95 were validated, and 16 of 20 randomly selected large indels were validated by PCR (Supplementary Fig. 11b-d, Supplementary Table 23). We also detected the sequence identities between each pair of homologous chromosomes with no gap alignment, and the sequence identity was 99.4% between these two haplotypes (Table 1).

Allelic and haplotype-specific genes were analyzed through synteny between two haplotypes (Fig. 3a). Among them, 14,685 pairs of genes with two alleles (having at least one amino acid difference) were identified, and 9,929 pairs had two copies of the same genotype. The average Ka/Ks ratio of these allele gene pairs was 0.46 (Fig. 3b), indicating that the majority of the allele genes underwent purifying selection. The number of Haplotype-specific genes included 1,653 in haplotype A and 2,061 in haplotype B (Table 1). Haplotype A-specific genes were enriched in the processes of response to other organisms, response to external biotic stimuli, and response to external stimuli, while Haplotype B-specific genes were enriched in nucleic acid phosphodiester bond hydrolysis, RNA modification, and RNA phosphodiester bond hydrolysis processes (Supplementary Fig. 14a and b). These results indicated that Haplotype A contributes more to the response to external environmental stimuli. Furthermore, it can be inferred that Haplotype A is likely to play a greater role in the two processes of leaf movement and nodule nitrogen fixation in *M. bimucronata*.

Based on the phased genome, we examined the expression differences between alleles and found that the expression level of the majority of the allele genes showed no significant differences between the two haplotypes (Fig. 3c). Interestingly, 930 gene pairs showed differential expression between the two alleles across the six tissues (Supplementary Table 24). Alleles with higher expression levels appeared randomly distributed between the two haplotypes and chromosomes, except for chromosomes 6, 10, and 13, which had fewer DEGs (Supplementary Table 24). GO analysis of the DEGs revealed enrichment in pathways that responded to biotic stimuli and external biotic stimuli in touch-closed leaves (TCL) and root (R) (Supplementary Fig. 15). Subsequently, these genes were further functionally annotated, uncovering that some genes might be involved in the processes of leaf movement or nodule nitrogen fixation (Supplementary Table 25). For instance, four genes, including *MbCML*, *MbTIP*, *MbNRT1*, and *MbGST,* with different expression patterns in the two haplotypes were predicted to work in the two different processes. *MbCML* and *MbTIP* might be involved in the process of leaf movement, while *MbNRT1* and *MbGST* could be associated with the process of nodule nitrogen fixation, which needs further investigation (Fig. 3d). The impact of SVs on allelic differences in gene expression was investigated by conducting an association analysis between SV-related genes and genes exhibiting allelic differential expression. Our findings revealed that 89 SV-related genes displayed differential expression between the two haplotypes (Fig. 3e, Supplementary Fig. 16), suggesting that SVs were involved in some important biological processes by influencing the differential expression of alleles.

### Analysis of key genes involved in leaf movement in *M. bimucronata*

Leaf movements in *M. bimucronata* include both seismogenic and nyctinastic movements. Transcriptome sequencing of *M. bimucronata* was performed to identify candidate genes involved in leaf movement. Three states of leaves were sequenced, including open leaves (OL) before touch, touch-closed leaves (TCL), and night-closed leaves (NCL) due to circadian clock regulation. Three biological replications for each sample were sequenced (Supplementary Table 26). Differentially expressed genes (DEGs) were identified from TCL vs. OL and NCL vs. OL with 574 DEGs and 3,336 DEGs, respectively (Supplementary Table 27).

**Fig. 4:**
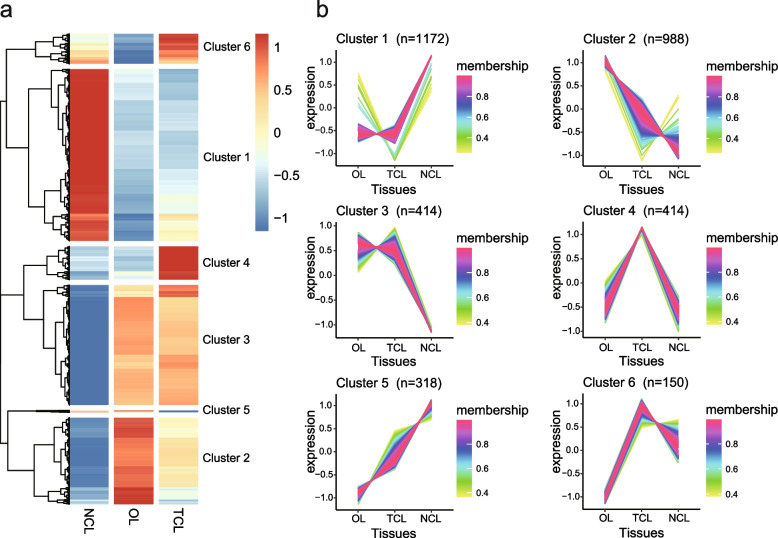
Different gene regulatory landscapes of differentially expressed genes of leaf movement. **a** Heatmap of differentially expressed genes in three different leaf stages (OL, NCL, TCL). Red indicates high expression, and blue indicates low expression. The average FPKM of three biological replicates was used as the expression level, and they were normalized by Log2 (FPKM + 1). **b** Six expression profile types of differentially expressed genes by TCseq analysis with fuzzy C-Means clustering. High affinity to the cluster centroid is shown in purple, and low affinity is shown in green

Expression clustering analysis of DEGs in three leaf states revealed 6 distinct expression profiles (Fig. 4a and 4b). Notably, the DEGs in Cluster 4 and Cluster 6 exhibited high expression levels in TCL, while the DEGs in Cluster 1 and Cluster 5 were highly expressed in NCL (Fig. 4b). Intriguingly, a set of genes in Cluster 2 was highly expressed in OL and expressed at low levels in TCL and NCL (Fig. 4b), corresponding to the open and closed states of leaves. Simultaneously, a set of genes in Cluster 3 was highly expressed in OL and TCL but expressed at low levels in NCL (Fig. 4b), which corresponded to leaves during the day and night.

GO and KEGG enrichment analyses were conducted on these DEGs in different expression profiles. For GO enrichment analysis, the DEGs from Cluster 4 and Cluster 6 were mainly involved in the biological processes of response to stimulus, response to stress, and response to oxygen-containing compounds (Supplementary Fig. 17a). The DEGs from Cluster 1 and Cluster 5 were mainly involved in the biological processes of regulation of RNA biosynthetic process, rhythmic process, and circadian rhythm (Supplementary Fig. 17b). The DEGs from Cluster 2 were enriched in the biological processes of ion transport, transmembrane transport, and inorganic ion transmembrane transport, while the DEGs from Cluster 3 were enriched in the biological processes of response to abiotic stimulus, response to light stimulus, and response to temperature stimulus (Supplementary Fig. 17c and d). For KEGG enrichment analysis, the DEGs from Cluster 4 and Cluster 6 were mainly involved in the biological pathways of biosynthesis of secondary metabolites, MAPK signaling pathway—plant and plant hormone signal transduction, while the DEGs from Cluster 1 and Cluster 5 were mainly involved in the biological pathways of plant hormone signal transduction, starch and sucrose metabolism and valine, leucine and isoleucine degradation (Supplementary Fig. 17b and 18a). The DEGs from Cluster 2 were enriched in the biological pathways of biosynthesis of secondary metabolites, plant hormone signal transduction, and circadian rhythm—plant, while the DEGs from Cluster 3 were enriched in the biological pathways of metabolic pathways, photosynthesis, and photosynthesis—antenna proteins (Supplementary Fig. 18c and d). Collectively, these results revealed that leaf movement is a complex process involving many biological processes and pathways. Seismonastic movement was mainly related to the biological process of stress response, whereas nyctinastic movement was mainly related to the biological process of rhythmic processes. Additionally, the processes of ion transmembrane transport and plant hormone signal transduction potentially played roles in both seismonastic and nyctinastic movements, while light and temperature also emerged as potential influencing factors on nyctinastic movement.

Combining functional annotation analysis and gene expression level considerations helped us identify the final candidate genes. For seismonastic movement, 57 candidate genes with high expression or expressed specifically in the touch-closed leaves were identified, mostly stress response genes, including zinc finger protein, ethylene-responsive transcription factor, and Nudix hydrolase (Supplementary Figs. 19c and 20a, Supplementary Table 27). For nyctinastic movement, 107 candidate genes with high expression or expressed specifically in the night-closed leaves were identified, including wound-induced protein, MIP aquaporin, universal stress protein, and the UDP-glycosyltransferase (Supplementary Figs. 19d and 20b, Supplementary Table 27).

The expression profile of DEGs from NCL vs. OL was investigated, revealing significant expression differences in 11 AQP genes, 12 Ca^2+^-related genes, and 20 ion channel genes (Supplementary Figs. 19a,b, and 21b). Additionally, two AQP genes, *Mb12g014040* and *Mb01g016320*, were highly expressed and upregulated in NCL compared to OL. Subsequently, 33 AQP genes in the genome of *M. bimucronata* were identified and a phylogenetic tree that included AQP genes from well-characterized species such as *A. thaliana* and *M. truncatula* was conducted. The *MbAQPs* were classified into 5 subfamilies (Supplementary Fig. 21a, Supplementary Table 28). The expression profile of all 33 *MbAQPs* showed that the members of the PIP and TIP subfamilies were expressed in almost all tissues (Supplementary Fig. 21b). Interestingly, both *Mb12g014040* and *Mb01g016320* belong to the PIP subfamily, while another upregulated gene, *Mb11g013010,* with a high expression level, belongs to the TIP subfamily.

### Analysis of gene coexpression networks of leaf movement in *M. bimucronata*

**Fig. 5:**
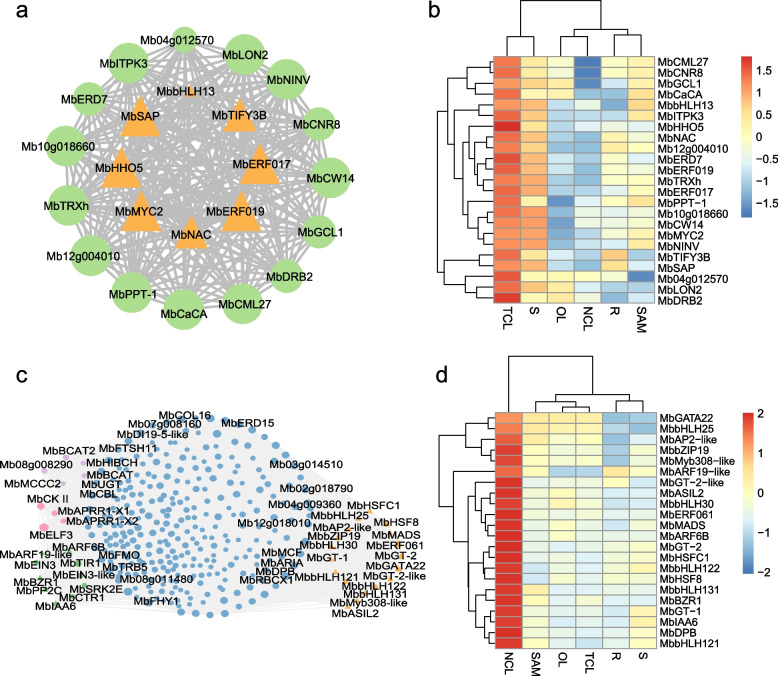
The coexpression network and expression profile of key genes involved in leaf movement. **a** The coexpression network of hub genes of the WGCNA “red” module related to TCL. Twenty-three genes with kME-p values greater than 0.9 were included in the Cytoscape-generated diagram. The orange triangle represents transcription factors, while the green circle represents some pathway genes. **b** Heatmap of the 23 genes, including 8 transcription factors and 15 pathway genes. Red indicates high expression, and blue indicates low expression. The average FPKM of three biological replicates was used as the expression level, and they were normalized by Log2 (FPKM + 1). **c** The coexpression network of hub genes of the WGCNA “yellow” module related to NCL. A total of 341 genes with kME-p values greater than 0.9 were included in the Cytoscape-generated diagram. The orange triangle represents transcription factors. The green diamonds represent genes involved in the plant hormone signal transduction pathway, the pink hexagons represent genes involved in the circadian rhythm pathway in plants, and the purple rectangles represent genes involved in the valine, leucine, and isoleucine degradation pathways. **d** Heatmap of the 22 transcription factor genes in the coexpression network of NCL. Red indicates high expression, and blue indicates low expression. The average FPKM of three biological replicates was used as the expression level, and they were normalized by Log2 (FPKM + 1)

To better understand the coexpression dynamics of genes involved in leaf movement in *M. bimucronata*, differential gene expression analysis was performed and a weighted gene coexpression network was conducted using weighted gene coexpression network analysis (WGCNA) based on the expressed genes. This analysis yielded 9 clusters (Supplementary Fig. 22). The module-tissue association analysis indicated that red and yellow modules were correlated with TCL and NCL, respectively (Supplementary Fig. 22). To identify the potentially important genes involved in leaf movement, module eigengene connectivity (kME) was calculated for each gene within the red and yellow modules. 23 and 341 genes in the red and yellow modules were identified as hub genes, respectively, and were used to construct the coexpression network (Fig. 5a and c, Supplementary Table 29 and 30). Within the coexpression network of TCL and NCL, 8 and 22 transcription factor (TF) genes were identified, respectively, belonging to different TF families. The heatmap displayed the expression profile of these TFs (Fig. 5b and d). Intriguingly, within the coexpression network of TCL, two genes, *MbCML27* (*Mb13g000970*) and *MbCaCA* (*Mb06g009710*), were identified, while in the coexpression network of NCL, one gene named *MbCBL* (*Mb04g013530*) was discovered. These genes could potentially play a vital role in leaf movement.

### Key nodulation gene analysis in *M. bimucronata*

**Fig. 6:**
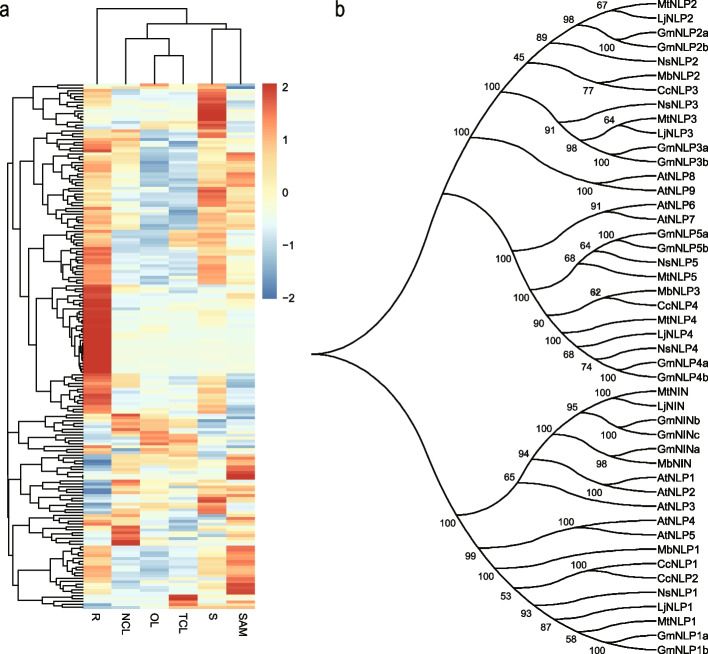
The expression file of nodulation-related genes and evolution analysis of NIN and NLP. **a** Heatmap of 189 homologous genes in *M. bimucronata.* Red indicates high expression, and blue indicates low expression. The average FPKM of three biological replicates was used as the expression level, and they were normalized by Log2 (FPKM + 1). **b** Phylogenetic tree analysis of NIN and NLP in seven species.

Rhizobial nodulation for nitrogen fixation is an important trait in most species in Fabaceae. The 30 key nodulation genes published in recent years were taken as queries, and a total of 189 orthologous genes in *M. bimucronata* were identified (Supplementary Table 31). The heatmap of the 189 candidate nodulation genes revealed that most of the genes were highly expressed in roots (Fig. 6a). Among the highly expressed genes, NIN (Nodule Inception), a transcription factor that is essential for nodulation, was selected for further analysis. We identified NIN and NIN-like protein (NLP) genes in *A. thaliana* and six other species, including four nodulation species (*L. japonicus*, *M. truncatula, G. max*, *and M. bimucronata*) and two non-nodulation species (*C. canadensis*, *N. schottii*) in Fabaceae and constructed the phylogenetic tree (Fig. 6b). The results revealed that a total of 46 NIN and NLP genes were identified in the seven species, all of which contained RWK-RK and PB1 domains (Supplementary Fig. 23). However, within the conserved region of NIN, a region that was not homologous to the corresponding regions of NLP was identified by multiple sequence alignment in *M. bimucronata* (Supplementary Fig. 24). Additionally, NIN was absent in the nonnodulation species, while NLP was found in both nodulation and nonnodulation species.

## Discussion

The availability of high-quality plant genome and pangenome assemblies facilitated studies of plant structural variations [35, 36]. Haplotype-resolved genomes make it possible to study SVs and allele specific expression (ASE) between haplotypes [37]. A recent study demonstrates that SVs can have an impact on gene expression and genotype-to-phenotype association on crop improvement [38]. In *M. bimucronata*, 714 SV-related genes were identified. GO enrichment revealed that these genes are primarily associated with processes of responses to herbivores, wounding, external biotic stimuli, and other organisms. This suggests that SVs might play a role in shaping the characteristics of leaf movement and rhizobial nodulation for nitrogen fixation in *M. bimucronata*.

The GO analysis of haplotype A- and haplotype B-specific genes revealed enrichment in distinct biological processes. The haplotype A genome might contribute more to the two typical features of leaf movement and nodule nitrogen fixation in the *M. bimucronata* genome. Additionally, GO analysis was conducted on ASE genes, leading to the identification of enrichment in biological processes of response to other organisms, biotic stimuli and external biotic stimuli, herbivore, and monoterpenoid biosynthetic and metabolic processes. These findings provide novel insights into the mechanisms governing leaf movement in *M. bimucronata*.

The integration of genomic and transcriptomic data provides valuable resources for gene discovery and facilitates the exploration of various aspects of plant growth, secondary metabolism, and physiological processes [39]. Among the DEGs identified, highly expressed and specifically expressed genes were selected as candidate genes involved in leaf movement. For seismonastic movement, the functions of candidate DEGs primarily involved transcription factors and cell-surface proteins. These genes likely mediate signal transduction and play regulatory roles in cell development, activation, growth and motility. For nyctinastic movement, in addition to transcription factors and cell-surface proteins, we also discovered genes associated with water transport and genes belonging to the plant dehydrin family. These genes are likely involved in regulating leaf movement through water flow in and out of cells. Furthermore, we predicted that genes acted in the modification of cell walls via demethylesterification of cell wall pectin in TCL vs. OL DEGs. Similarly, genes related to tubulin and cell wall construction were predicted in NCL vs. OL DEGs. These findings align with previous research showing that actin phosphorylation at tyrosine residues can impact actin filament reorganization, resulting in seismonastic movement in *M. pudica* [40, 41]. Additionally, alteration in secondary cell wall biosynthesis can affect the physical strength of the lamina joints, leading to changes in the angle of the flag leaf blade in rice [42], suggesting that some genes regulating cell wall formation play a vital role in leaf movement in *M. bimucronata*.

Leaf movement is a complex process that encompasses various genes, biological processes, and pathways, serving as a plant's response to external stimuli [43, 44]. Ca^2+^ has been recognized as a crucial messenger, playing a vital role in plant stress responses through calcium-dependent signaling pathways [11, 45]. In plant cells, three main types of sensors receive Ca^2+^ signals, including calmodulin (CaM/CAM) and CaM-like (CML) proteins, calcium-dependent protein kinases (CDPKs/CPKs), calcineurin B-like proteins (CBL) and CBL-interacting protein kinases (CIPKs) [46]. Among them, CaM and CMLs function as signal molecules in signal transduction and response to environmental stimuli. Notably, *GsCML27*, a Ca^2+^-binding EF-hand protein, has been demonstrated to play a role in plant responses to bicarbonate, salt, and osmotic stresses [47].

Transporters play a crucial role in the transport of Ca^2+^ and other cations and are essential for mineral nutrition, ion stress tolerance, and signal transduction [48]. Previous studies have unveiled emerging vital roles of CBL proteins in plant abiotic stress tolerance [49, 50]. These discoveries emphasized the potential involvement of Ca^2+^ in signal transduction and plant stress resistance. We identified a calcium-binding protein, *MbCML27* (*Mb13g000970*), and a transporter that belongs to the Ca (2 +) cation antiporter (CaCA) (TC 2.A.19) family, *MbCaCA* (*Mb06g009710*), in the coexpression network of TCL. This finding suggested the potential contributions of Ca^2+^ and cation transporters to leaf movement. We also identified a calcineurin B-like protein, *MbCBL* (*Mb04g013530*), a Ca^2+^ sensor, in the coexpression network of NCL. This discovery indicated that Ca^2+^ sensors might perceive external stimuli, translate the signal, and potentially participate in leaf movement. Additionally, AQP (aquaporin) genes were identified in the gene coexpression network of TCL and NCL tissues. Collectively, these findings lead us to speculate that AQP genes and calcium signaling might serve as important regulators of both leaf movement and nodule nitrogen fixation.

Rhizobial nodulation for nitrogen fixation is a crucial trait shared by most species in the Fabaceae family, with 92.27% of Fabaceae species exhibiting the trait [25]. This high prevalence of rhizobial nodulation has been one of the reasons that contributed to the enhancement of species diversity, making Fabaceae the third largest angiosperm family [25]. Building upon the knowledge of previously published 30 key nodulation genes, we identified 189 homologous genes of these key nodulation genes in *M. bimucronata*. Through evolutionary analysis, we investigated the relationship between *NIN* and *NLP* genes in nodulation and nonnodulation species, confirming that *NIN* is lost in nonnodulation species [25, 32]. Furthermore, multiple sequence alignments revealed differences in the N-terminal region of NIN and NLP, suggesting variation in their functions. A previous study in *L. japonicus* demonstrates that the N-terminal region of NIN does not respond to nitrate, leading to the hypothesis that the loss of nitrate responsiveness in NIN might be necessary for the emergence of symbiotic nitrogen fixation in Fabaceae [51]. This adaptation allows for the induction of root nodule formation under nitrogen-deficient conditions, a characteristic of symbiotic nitrogen fixation [51]. Conserved domain analysis uncovered the presence of the RWK-RK and PB1 domains in all NIN and NLP genes, highlighting their close relationship and supporting the notion that NIN serves as the founding member of the NLP family [52].

Leaf nyctinastic movement and nodule nitrogen fixation are prominent features in the majority of Fabaceae species, representing two energy-consuming processes, while leaf seismonastic movement mainly exists in some clades of *Mimosa* [2]. This preservation underscores the significance of these traits and suggests their essentiality in enabling legumes to adapt to their environment. Leaf seismonastic movement is hypothesized to have evolved independently in eight lineages of *Mimosa,* and that *M. pudica* and *M. bimucronata* were found in distant clades [2], suggesting that the trait has a different genomic underpinning in these two species. A recent study has demonstrated that *M. pudica* is a tetraploid plant and the genome has been published [24]. Our study indicated that *M. bimucronata* was a diploid species and the genome was assembled. Combined with the published genome of *M. pudica,* the new genome provided new insights to improve the understanding of leaf movement in *Mimosa* from a genomic and comparative genomics perspective. Furthermore, the dosage effect caused by different chromosome ploidy could be the cause of the difference in leaf movement sensitivity to external stimuli. Recent research focusing on the clock gene LHY in *M. truncatula* has shed light on its role in nodulation. Loss of *MtLHY* function is found to reduce nodule formation and impair nitrogen assimilation, consequently affecting the endogenous circadian rhythm in nodules and ultimately impacting nyctinastic leaf movement and biomass reduction [53], establishing a correlation between leaf nyctinastic movement and nodule nitrogen fixation.

## Conclusion

We have reported a high-quality, haplotype-resolved, chromosome-level genome of *M. bimucronata*, offering a valuable genetic resource for further investigations and serving as a key reference for comparative genomics research of legumes. Through structural variants analysis and allele-specific gene expression studies based on the haplotype-resolved assembly, we have unveiled the potential role of structural variants on leaf movement and nitrogen fixation in *M. bimucronata*. Furthermore, the detailed transcriptome analysis has identified numerous promising candidate genes, making it possible to uncover the molecular mechanism of plant leaf movement and nitrogen fixation at the gene level.

## Materials and methods

### Plant materials and growth conditions

Seeds of *M. bimucronata* were obtained from the Germplasm Bank of Wild Species in Southwest China (http://www.genobank.org/). To promote germination, the seeds underwent a brief treatment with 95 °C hot water for one minute, followed by placement in a petri dish with filter paper. Subsequently, the germinated seeds were carefully planted in a greenhouse environment maintained at 25 °C with a 16/8 h light–dark photoperiod cycle.

### The statistics of chromosome number and ploidy evaluation

Plants of *M. bimucronata* were cultivated in the greenhouse for approximately one month before the root tip was fixed for chromosome number analysis. We followed the method outlined by Xin et al. [54] with slight adjustments to adapt to our plant samples. The enzymatic digestion process involved treating the roots with a mixture comprising 4% cellulose, 2% pectinase, and 1% pectolyase dissolved in 0.01 M citrate buffer (pH 4.5) at 37 °C, with the digestion time reduced from 1 h to 30 min. The well-spread mitotic chromosome preparations were selected and stained with 1.5 μg/mL 4,6-diamidino-2-phenylindole (DAPI) and then used for chromosome number observation. Images of chromosome spreads were captured using a Lecia microscope.

Smudgeplot (v0.2.2) was used for ploidy estimation based on the K-mer analysis [55].

### DNA extraction, library construction, and genome sequencing

Fresh and healthy leaves were promptly collected and flash-frozen in liquid nitrogen. Subsequently, these frozen samples were sent to the biotechnology company BioMarker with dry ice during transit. High-quality genomic DNA was extracted from *M. bimucronata* leaves using the cetyltrimethylammonium bromide (CTAB) method [56]. The quality and quantity of the isolated DNA were assessed by Qubit2.0 Fluorometer. To facilitate comprehensive genomic analysis, PacBio HiFi (CCS), Illumina, and Hi-C libraries were constructed following the instructions for each technology. For the PacBio HiFi library, the whole genome was sequenced on the PacBio Sequel II system based on a single-molecule, real-time (SMRT) sequencing approach. The Illumina library was sequenced on the Illumina HiSeq X Ten platform following the standard protocol provided by Illumina with an insert size of 350 bp and the paired ends in 150 (PE150) mode. The Hi-C library was sequenced on the same platform as the Illumina data, using PE150 mode.

### Estimation of genome size by FCM and the K-mer method

The molecular biology experimental platform of the Southwest Wildlife Germplasm Bank completed the estimation of the genome size of *M. bimucronata* using flow cytometry (FCM), using *Zea mays* as the internal reference with a known genome size of 2,300 Mb.

GCE (v1.0.2) was employed to calculate the K-mer depth and frequency of the genome, allowing for the precise estimation of genome size and heterozygosity [57]. Subsequently, the R package was applied for K-mer histogram analysis and mapping of the K-mer distribution. enhancing the comprehensive understanding of *M. bimucronata*'s genomic characteristics.

### Genome assembly and quality assessment

The genome was assembled using HiFiasm (v0.15.5) [58], with subsequent removal of redundancy in the preliminary genome assembly achieved through Khaper (https://github.com/lardo/khaper). The consensus genome was polished by NextPolish (v1.3.1) with Illumina data [59]. For contig anchoring to chromosomes and refinement to achieve a chromosome-level genome, ALLHi-C was employed for anchoring the contigs to the chromosomes, and the JUICEBOX tool was used to perform the assembly error correction [60, 61]. Ragtag (v2.1.0) was applied for scaffolding the haplotype-resolved genome with the monoploid genome as a reference [62]. All the above mentioned tools were performed with default parameters.

BUSCO (v3.0.2) with OrthoDB (embryophyta_odb10) was used to evaluate the quality of the final genome [63, 64]. To confirm the quality of the monoploid genome, Illumina data and RNA-sequencing data were mapped to the final genome with BWA-MEM (0.7.13-r1126) [65]. The LTR assembly index (LAI) was also used to further assess the quality of the genome assemblies, including monoploid and Haplotype-resolved genomes [66]. Merqury (v1.3) was used for further quality assessment of the genome assemblies based on K-mer analysis [67].

### RNA extraction, library construction, RNA sequencing, and analysis

Total RNA was extracted from different tissues, including open leaves, touch-closed leaves, night-closed leaves, stems (S), roots, and stem apical meristems (SAM) using a Polysaccharide Polyphenol Plant Total RNA Extraction Kit (TIANGEN, DP441) according to the manufacturer’s instructions. Each sample had three biological replicates. The isolated RNA was subjected to perform quality assessment using NanoDrop and Qubit2.0 Fluorometer. Subsequently, high-quality RNA was sent to Berry Genomics Company for library construction and sequencing.

The sequencing reads were aligned to the assembled genome using HISAT2 [68]. Stringtie2 [69] was applied to calculate the expression levels of all genes through the fragments per kilobase of transcript per million fragments mapped (FPKM). DeSeq2 [70] was employed for the identification of significant Differentially Expressed Genes (DEGs), with a False Discovery Rate (FDR) ≤ 0.05 and an absolute log2 (Fold Change) ≥ 1 set as the threshold.

### Gene prediction, structural and functional annotation

GETA, an automatic genome-wide annotation tool with improved accuracy and gene integrity for eukaryotes that integrates three methods, including homology-based methods, de novo methods, and transcriptome-based methods, was used to predict the protein-coding genes of the *M. bimucronata* genome (https://github.com/chenlianfu/geta). BUSCO (v3.0.2) was used for the evaluation of annotation completeness [63, 64].

To identify non-coding RNA elements, rRNA, miRNA, and snRNA were predicted using INFERNAL (v1.1.4) based on the Rfam database with default parameters [71]. tRNAscan-SE (v2.0.9) was applied to identify tRNA [72].

Repetitive sequences of the *M. bimucronata* genome were annotated with RepeatMasker (v4.0.9) [73] and RepeatModeler (v1.0.8) [74] against the RepBase database [75]. TEclass was used to classify the unknown repeat sequences [76], while LTR_FINDER was used to identify intact LTR retrotransposons, and Tandem Repeats Finder (TRF) was used to detect tandem repeat sequences [77, 78].

Functional annotation of the protein-coding genes was performed with EggNOGmapper software online with the default parameters [79, 80]. Additionally, a BLASTP (E-value = 1e-5) search was conducted against UniProt/SwissProt [81], the Nonredundant Protein Sequence Database (NR), Clusters of Orthologous Groups for Eukaryotic Complete Genome (KOG) [82] and Kyoto Encyclopedia of Genes and Genomes (KEGG) databases [83].

### Detection of structural variations and analysis of allele gene expression

Whole-genome alignments of the chromosome pairs in two haplotypes were performed using the Nucmer alignment tool with the parameters -c 500 -b 500 -l 50. The subprogram delta-filter was performed to filter the alignments with parameters -i 90 and -l 100 and show-coords was conducted to convert the alignments tab-delimited files with default parameters [84]. Subsequently, SYRI (v1.6) [85] was employed to detect the structural variations and the distributions of structural variations were visualized by circos (v0.69–8) [86] and plotsr (v0.5.4) [87]. The subprogram show-snps was applied for identifying the SNPs, and Assemblytics (v1.2) was used to identify INDELs based on the alignments from Nucmer. The distribution of SNPs and INDELs on the chromosome was depicted using the RIdeogram package [88].

For generating high-quality SVs, inversions were manually checked using a method referencing SV detection in the marmoset diploid genome [89]. The method was refined through the following steps: (1) Trimming 2000 bp of upstream/downstream flanking sequences of each breakpoint between the two haplotypes; (2) Aligning local PacBio HiFi reads and Illumina reads to the breakpoint with flanking sequences using minimap2 (v2.26-r1175) [90] and BWA-MEM (v0.7.13-r1126) [65], respectively; (3) Visualizing the alignments of PacBio reads and Illumina reads using the Integrative Genomics Viewer (IGV) [91]. Additionally, 100 large indels on chromosome 1 were randomly selected by the code published on GitHub (https://github.com/comery/marmoset) and were checked with PacBio long reads to ensure accuracy. Sequence identity between pairs of homologous chromosomes without gaps in alignment blocks was compared, following the analysis method of haplotype variations at the genome level in the tea genome. Sequence identities between pairs of homologous chromosomes without gaps in alignment blocks were compared, following the analysis method of haplotype variations at the genome level in the tea genome [37].

To identify allelic genes, the method of haplotype comparison and diversity analysis in the tea genome [37] was referenced and the code published on GitHub (https://github.com/sc-zhang/AlleleFinder/) was used.

### Gene coexpression network analysis

To explore the relationship between genes involved in leaf movement, weighted gene coexpression network analysis (WGCNA) with the WGCNA (v 1.71) [92] package in R was conducted. Expression data were prefiltered with the standard that a gene was considered expressed when the FPKM was exceeded 3 in at least one tissue. The eigengene value was calculated for each module and used to test the association with each tissue type to perform the module-tissue association analysis. Total connectivity and intramodular connectivity, kME (Pearson correlation between gene expression level and modular membership), and kME-p value were calculated. For hub gene selection, a kME-p value exceeding 0.9 served as the threshold, ultimately leading to the identification of tissue-specific modules. The resulting network was visualized using Cytoscape (v3.9.1) a kME-p value exceeding 0.9 was taken as the threshold, ultimately resulting in the identification of tissue-specific modules. Finally, Cytoscape (v3.9.1) [93] was used to display the network.

### Gene family and phylogenetic analysis

Orthofinder (v2.3.3) [94] with default parameters was used to identify and cluster gene families from *M. bimucronata* and seven other species, including *F. albida*, *S. tora*, *D. odorifera*, *M. truncatula*, *A. thaliana*, *C. papaya*, and *O. sativa.* The genome sequences and protein sequences were downloaded from public databases (Supplementary Table 17).

Single-copy orthologous genes identified by Orthofinder (v2.3.3) results were extracted, and the corresponding protein sequences were aligned by MAFFT (v7.307) [95]. Subsequently, a maximum-likelihood phylogenetic tree was constructed using RaxML (v8.2.12) based on the protein alignments [96]. The MCMCTree program within the PAML package [97] was employed to estimate the species divergence times among the eight species with the following main parameters: burn-in = 2,000, sample number = 20,000, and sample frequency = 2. Additionally, one calibration point was the divergence time (68–72 million years ago) between *A. thaliana* and *C. papaya* and the other was the monocot and eudicot divergence time (120–140 million years ago), which was shown by *A. thaliana* and *O. sativa* [98].

### Gene family expansion/contraction analysis and species-specific gene distribution on chromosomes in *M. bimucronata*

CAFE (v4.2.1) [99] was used to identify the gene families that undergoing expansion or contraction in the eight sequenced species, with a significance threshold set at *P* < 0.05. The rapidly evolving gene families in *M. bimucronata* were extracted and subsequently analyzed for GO enrichment and KEGG pathway analysis using OmicShare (https://www.omicshare.com/). Simultaneously, species-specific genes in *M. bimucronata* were also extracted and subjected to GO enrichment analysis and KEGG pathway analysis on the OmicShare platform. The distribution of these species-specific genes on the chromosomes was displayed by TBtools [100]. All GO and KEGG enrichments in subsequent analyses were conducted through the OmicShare platform.

### WGD events and collinearity

Both *M. bimucronata* and *S. tora* belong to the subfamily Caesalpinioideae of the legume family and share the same basic number of chromosomes (x = 13). To explore the evolutionary dynamics of the *M. bimucronata* genome, the WGD pipeline was used to calculate the distribution of synonymous substitutions per synonymous site (Ks), aiming to identify potential WGD events [101]. Based on the value of 5.17 × 10^−3^ and the formula of T = K/2r (where K represents the number of substitutions per base between genomes), the divergence time was calculated [102]. For comparative genome analyses of *M. bimucronata* and *S. tora*, MCScanX was used to identify and visualize the syntenic blocks [103].

### Investigation of the AQP gene family

Three methods, including extraction from the annotation of the *M. bimucronata* genome, based on the HMM profile of AQPs (PF00230), and based on BLASTP, were conducted to identify the AQP gene family. In addition, the protein sequences of the AQP gene families of Arabidopsis and Medicago were downloaded from the Phytozome database (https://phytozome.jgi.doe.gov/pz/portal.html). Subsequently, ClustalW was used for multiple sequence alignment, and MEGA-X [104] was performed in neighbor-joining mode with 1000 bootstrap replicates. The phylogenetic tree was visualized with an online tool, namely, Evolview (https://evolgenius.info//evolview-v2/#login).

### Key nodulation gene identification in *M. bimucronata*

The 30 key nodulation-related genes that can be obtained from the public (https://github.com/Genomic-docker/Evolution-of-key-nodulaiton-genes) were utilized as the queries [25]. To identify the homologs genes of these key nodulation-related genes in the *M. bimucronata* genome, a BLASTP search with a threshold of less than 1e-5 and at least 20% amino acid sequence identification was conducted. The heatmap of these homolog genes was displayed by R. The protein sequences of NIN (Nodule Inception) and NLP (NIN-like Protein) genes from nodulation and nonnodulation species were identified. A phylogenetic tree was constructed using MEGA-X with neighbor-joining mode and with 1000 bootstrap replicates [104].

### Supplementary Information

**Supplementary Material 1.**

**Supplementary Material 2.**

**Supplementary Material 3.**

**Supplementary Material 4.**

**Supplementary Material 5.**

**Supplementary Material 6.**

## Data Availability

The raw sequencing data of Pacbio HiFi reads, HiC reads and RNA-seq reads, as well as genome assemblies and annotations have been deposited at the National Genomics Data Center (https://ngdc.cncb.ac.cn) under BioProject PRJCA018116. The raw DNA and RNA sequence data were reported under accession numbers CRA011718 and CRA011721 that are publicly accessible at https://ngdc.cncb.ac.cn/gsa, respectively. The whole-genome sequence data was reported under accession number GWHDODZ00000000 and is publicly accessible at https://ngdc.cncb.ac.cn/gwh.

## Abbreviations

AQPs: Aquaporins
WGD: Whole Genome Duplication
SNF: Symbiotic Nitrogen Fixation
SVs: Structural Variations
DUPs: Duplications
INVs: Inversions
INVDPs: Inverted duplications
INVTRs: Inverted translocations
TRANSs: Translocations
OL Open: Leaves
TCL: Touch-closed leaves
NCL: Night-closed leaves
DEGs: Differentially expressed genes
CML: Calmodulin-like
XTH: Xyloglucan endotransglucosylase/hydrolase
NIN: Nodule Inception
NLP: NIN-like Protein
LTR: Long terminal repeat retrotransposons
LAI: LTR Assembly Index
WGCNA: Weighted gene coexpression network analysis
ASE: Allele specific expression
FPKM: Fragments per kilobase of transcript per million fragments mapped
