# Supplementary material for: Haplotype-resolved genome of Mimosa bimucronata revealed insights into leaf movement and nitrogen fixation
Source: BMC Genomics. 2024 Apr 3;25:334. doi: 10.1186/s12864-024-10264-8 (PMC10993578; doi:10.1186/s12864-024-10264-8)
Supplement: Supplementary file 1 — Supplementary Material 1. [file 12864_2024_10264_MOESM1_ESM.doc]

**Haplotype-resolved genome of *Mimosa bimucronata* revealed insights into leaf movement** **and nitrogen fixation**

Haifeng Jia^1^ , Jishan Lin^2^, Zhicong Lin^3^ , Yibin Wang^4^, Liangwei Xu^1,5^, Wenjie Ding^1,5^ and Ray Ming^1*^

Supplementary Figures


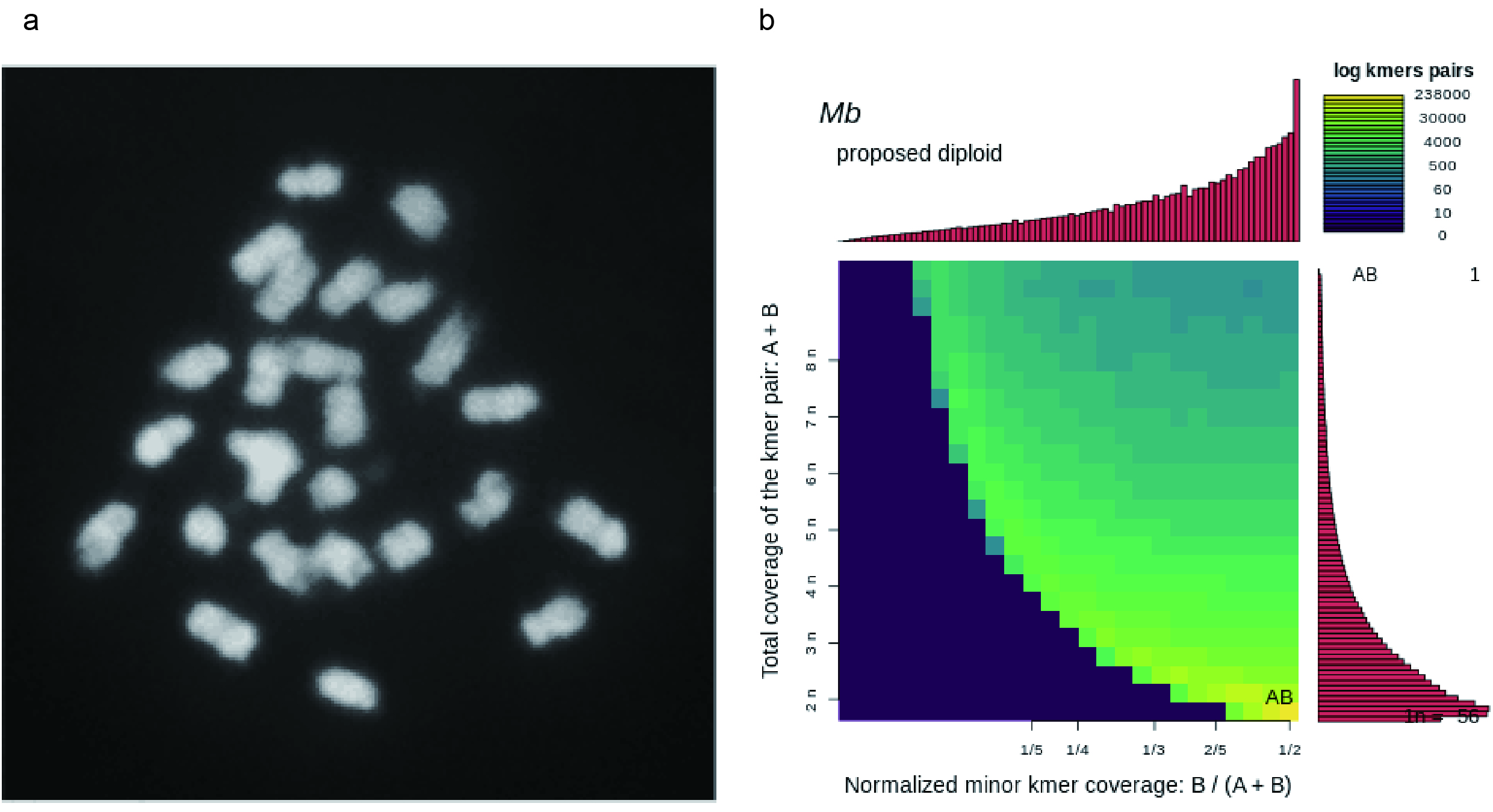


1. **Supplementary Figure 1.** [Karyotype](javascript:;) analysis and ploid estimation of *M. bimucronata.*
2. **a** [Karyotype](javascript:;) of *M. bimucronata* (2n = 26).
3. **b** Ploid estimation by Smudgeplot.


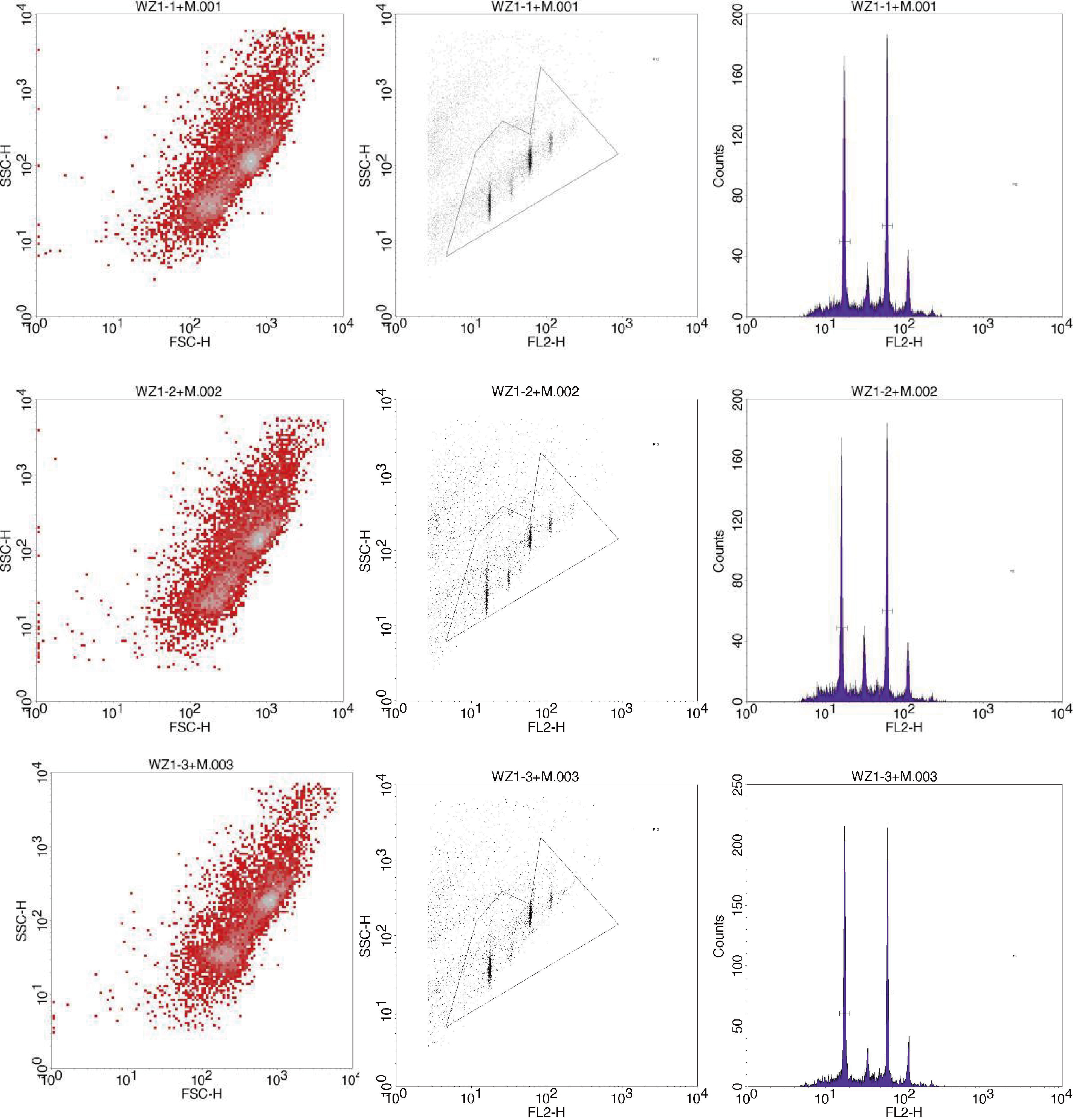


**Supplementary Figure 2.** Histogram of FCM results of *M. bimucronata* with three biological

repetitions.


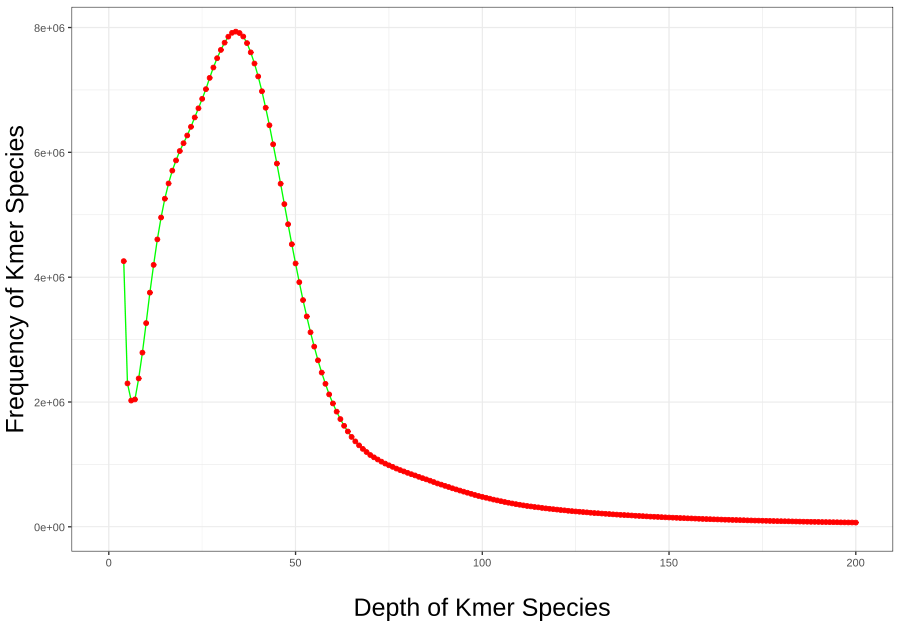


**Supplementary Figure 3.** Plots for *M. bimucronata* of K-mer 17.


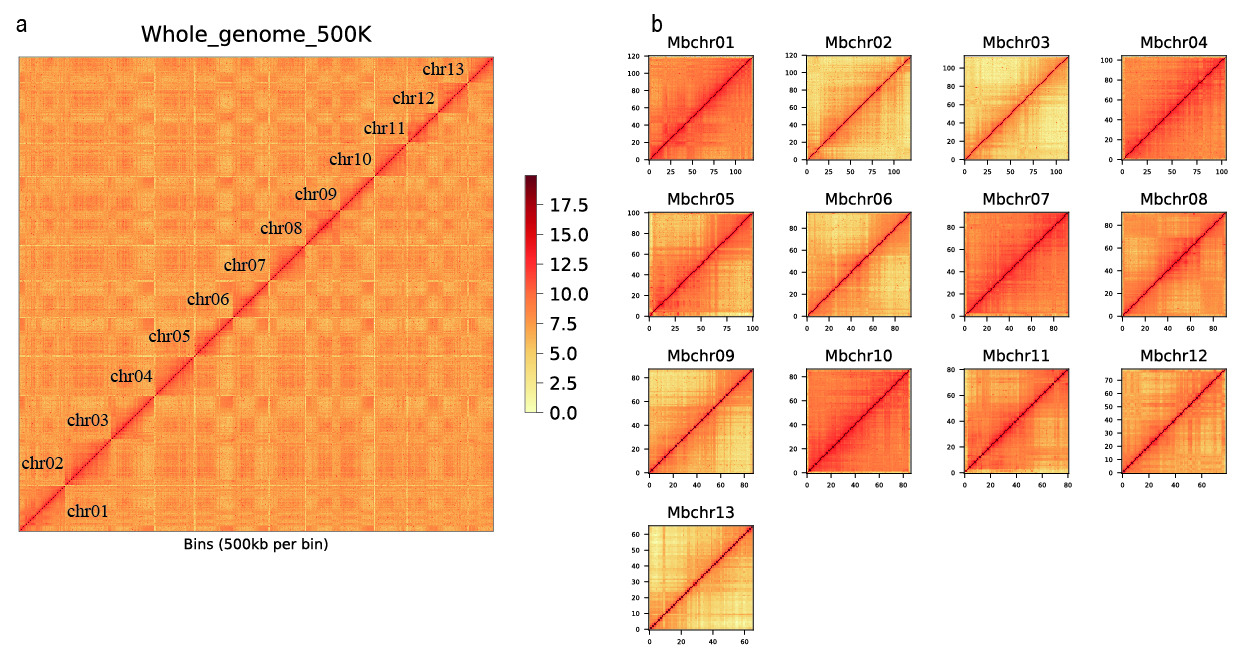


**Supplementary Figure 4.** Hi-C interaction heat map.

**a** The Hi-C interaction heatmap plot of *M.bimucronata* genome.

**b** Hi-C assistant mapping of 13 chromosomes of the *M. bimucronata* genome. Hi-C heatmaps are shown at 500 kb resolution.


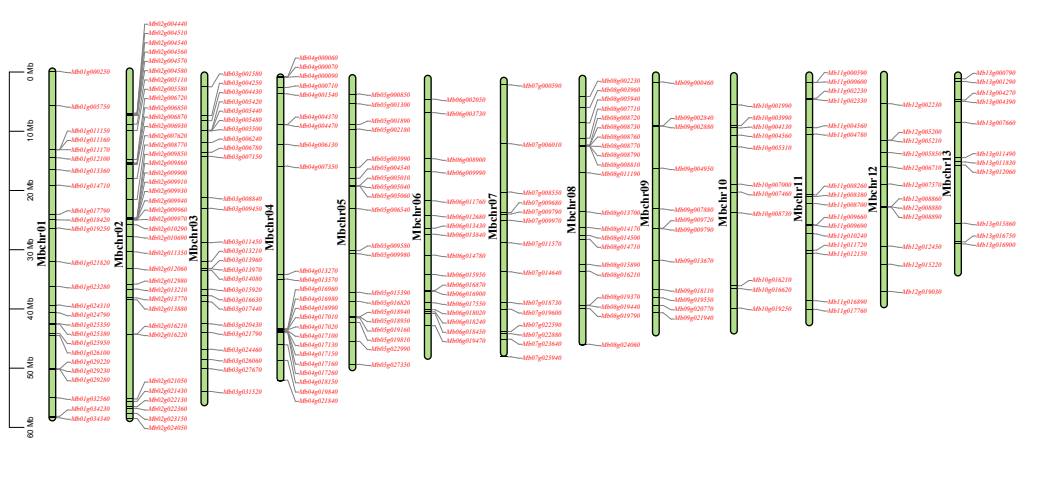


**Supplementary Figure 5.** Distribution of species-specific genes on 13 chromosomes.


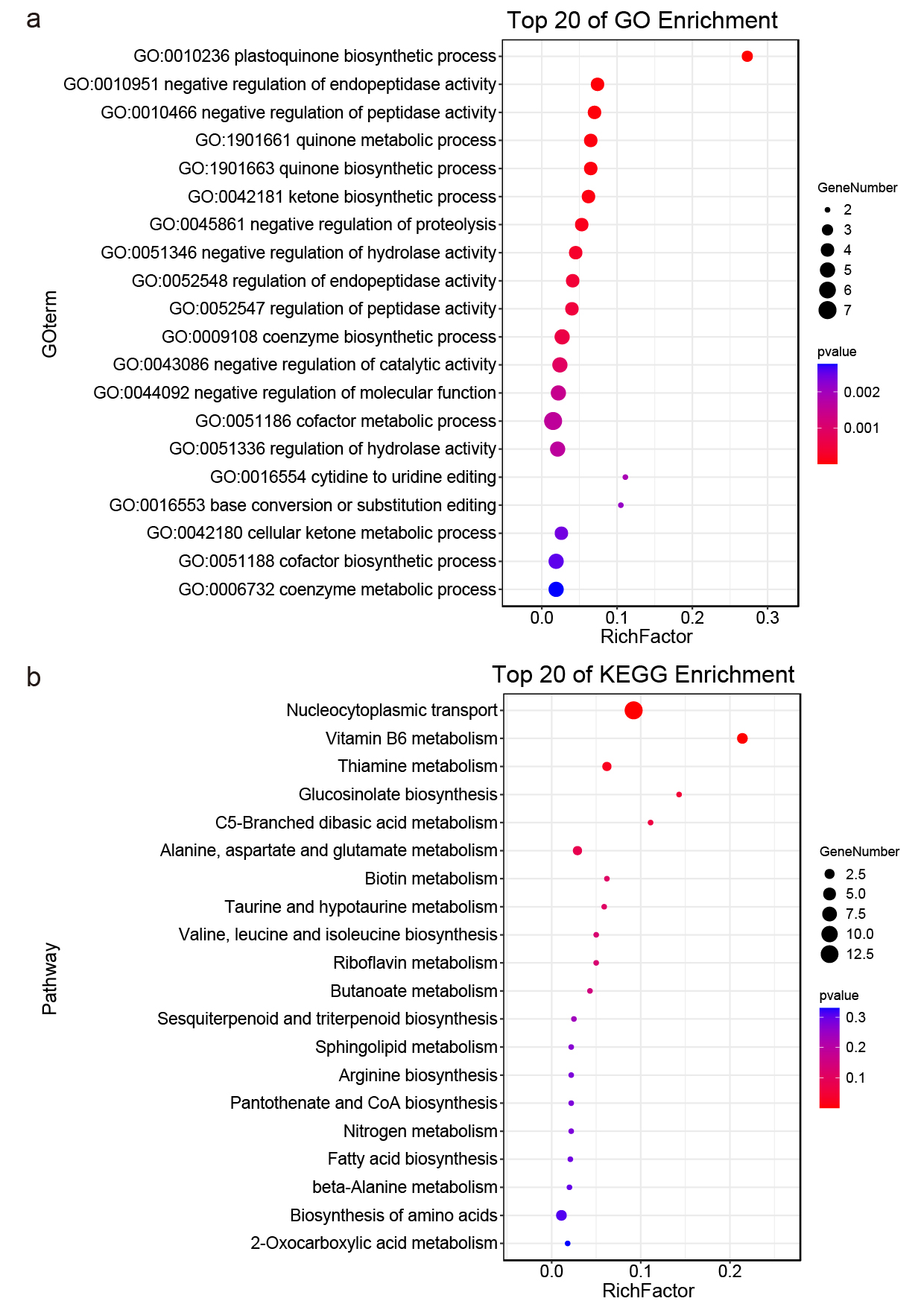


**Supplementary Figure 6.** GO terms and KEGG pathways of species-specific genes of *M. bimucronata* (a GO enrichment b KEGG enrichment).


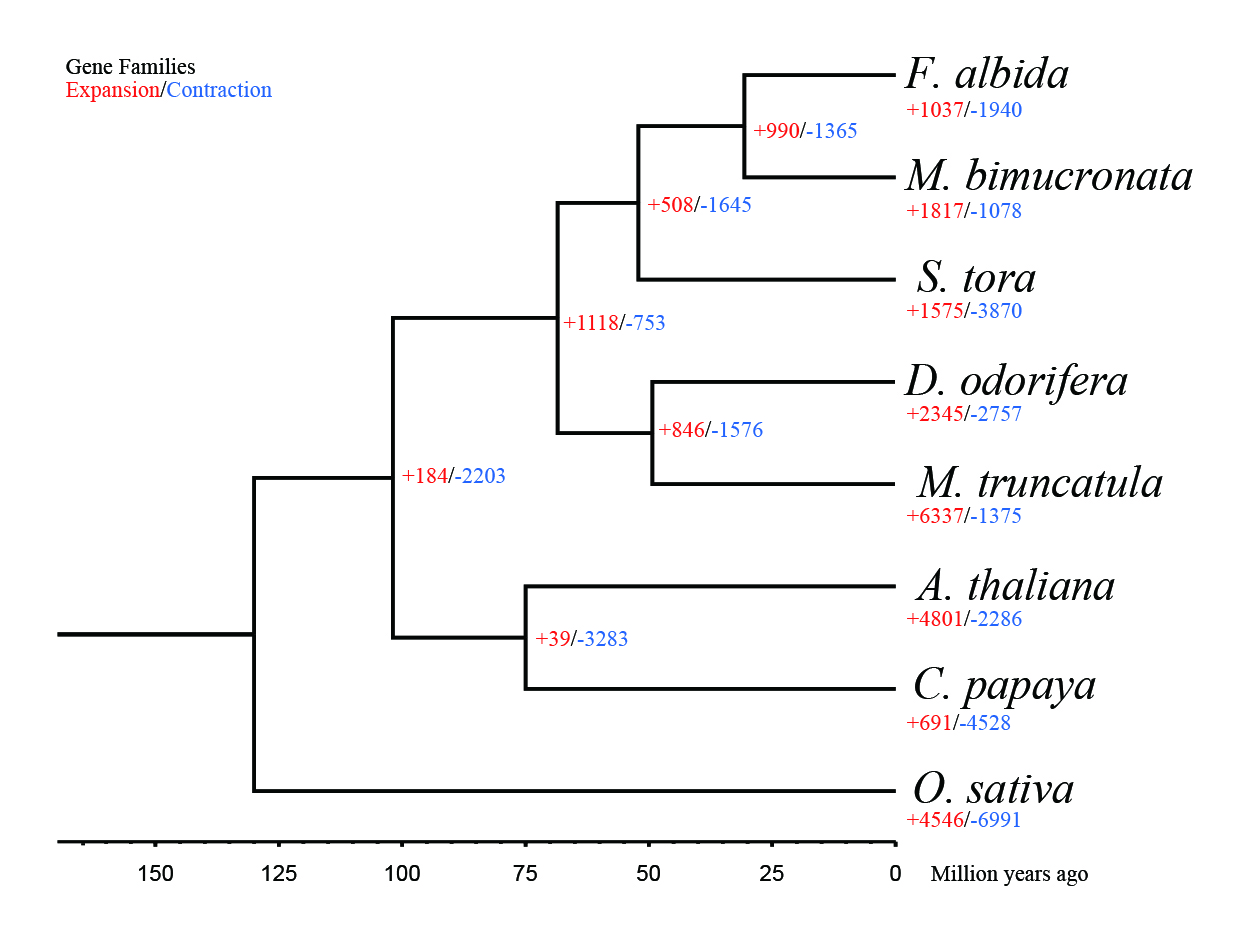


**Supplementary Figure 7.** Gene families expansion/contraction of 8 species.

The red and blue numbers indicate gene families of expansion and contraction, respectively.


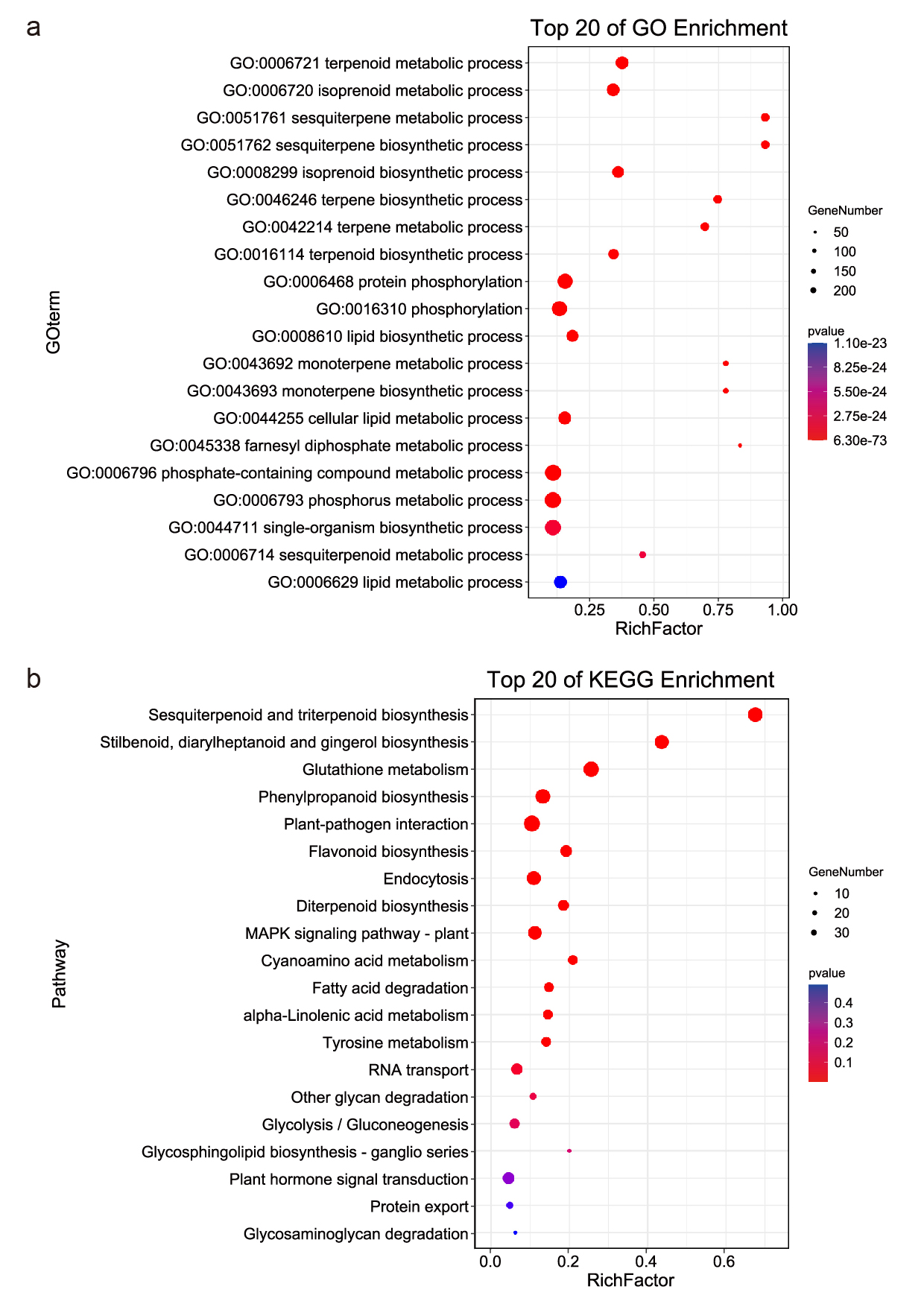


**Supplementary Figure 8.** GO terms and KEGG pathways of rapidly evolving gene families of *M. bimucrona* (a GO enrichment b KEGG enrichment).


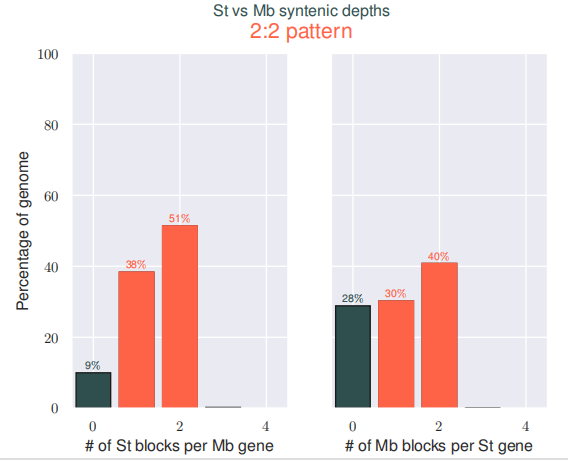


**Supplementary Figure 9.** *S. tora* and *M. bimucronata* syntenic depth.


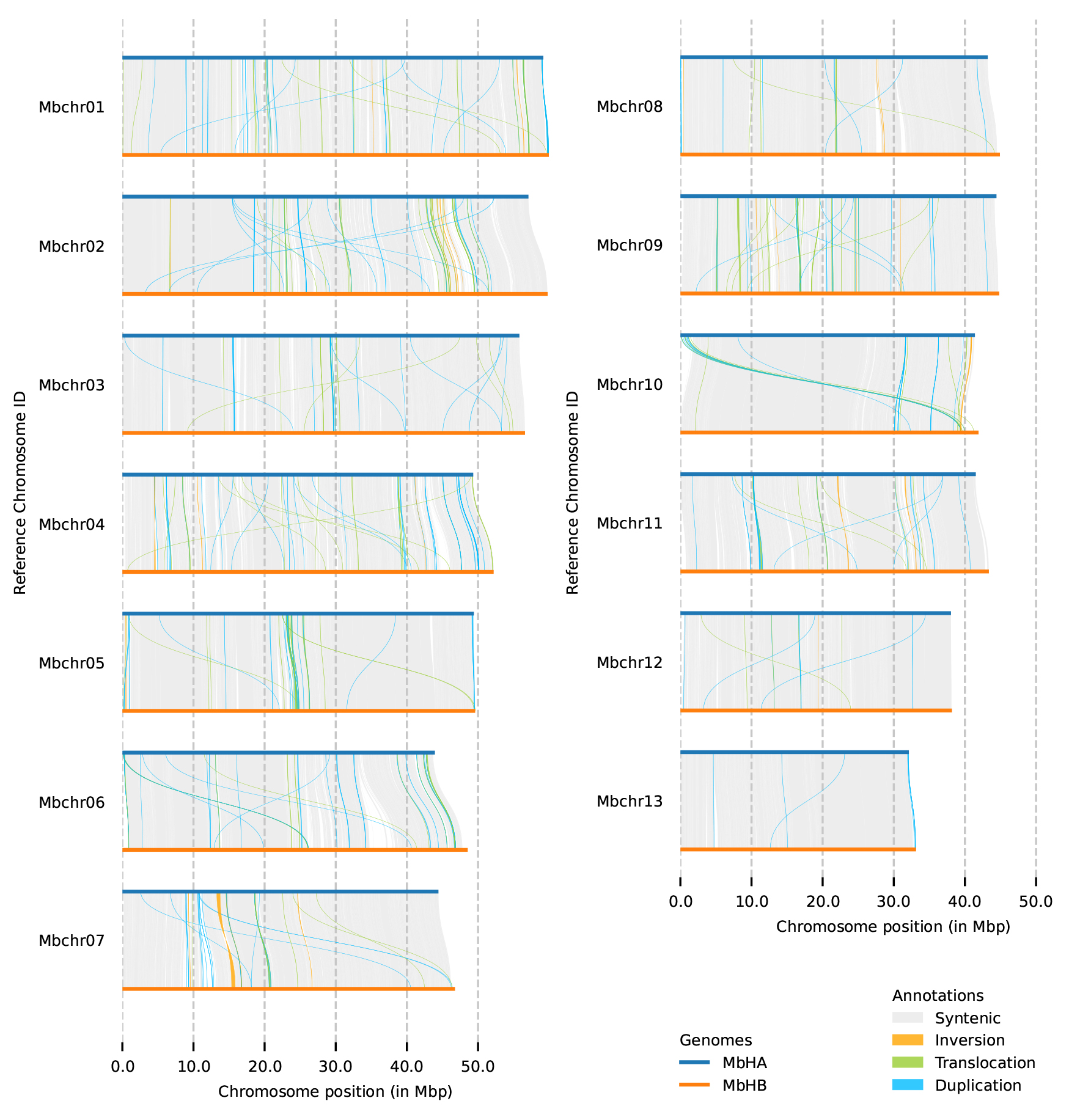


**Supplementary Figure 10.** Visualization of structural variation between homologous chromosome pairs.

**
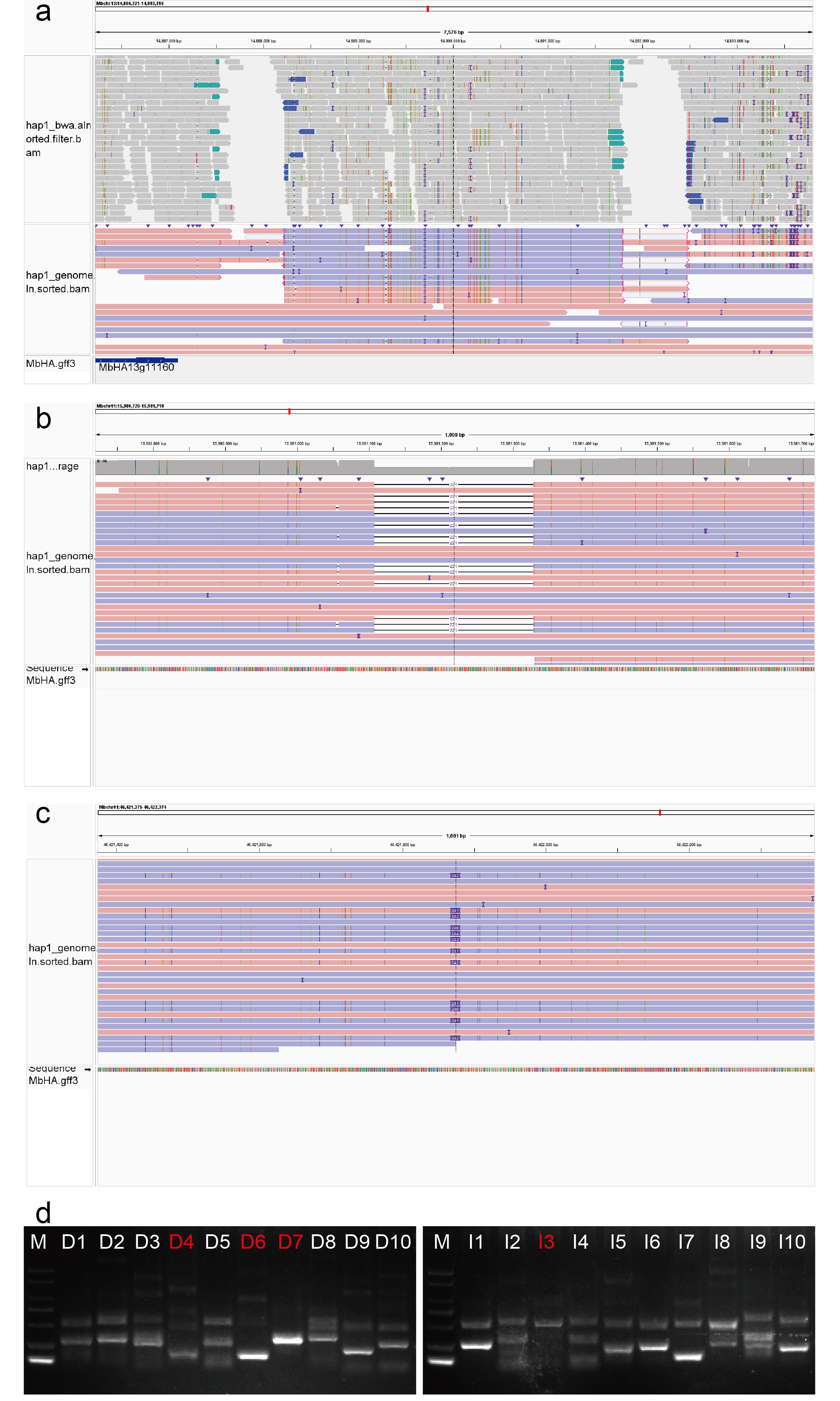
**

**Supplementary Figure 11.** Visualization of structural variations between the genomes of two haplotypes. **a** An example of inversion verified by Illumina data and PacBio HiFi long reads. **b** An example of deletion verified by Pacbio HiFi long reads. **c** An example of insertion verified by PacBio HiFi long reads. **d** PCR validation of 20 randomly selected large indels on chromosome 1. The seven gel bands on the left are length of 2 kb, 1.5kb, 1 kb, 750 bp, 500 bp, 250 bp, and 100 bp, respectively. The white color represents the validation success whereas red represents failure.


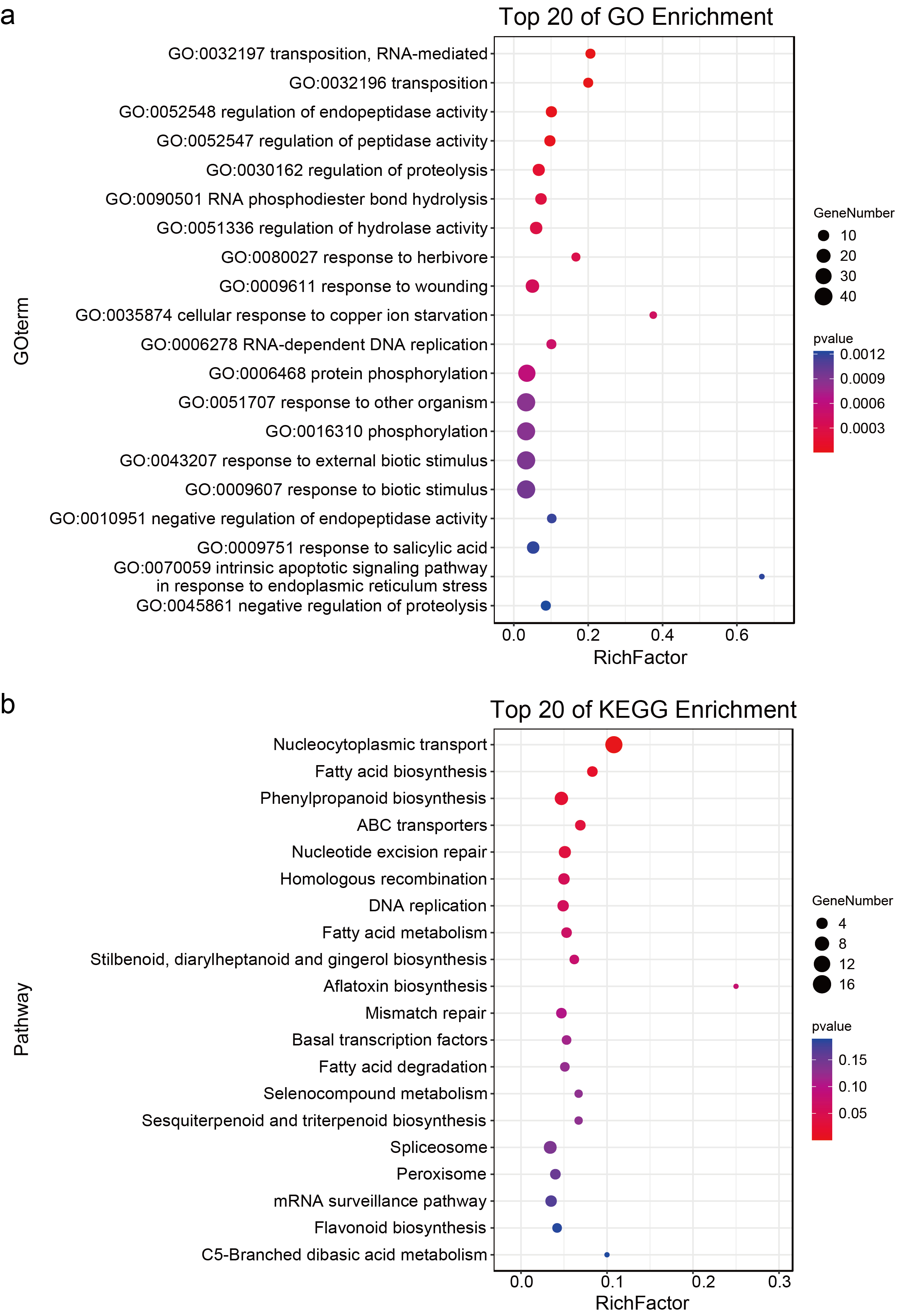


**Supplementary Figure 12.** GO and KEGG analysis of SV-related genes.

(**a** GO analysis **b** KEGG analysis)


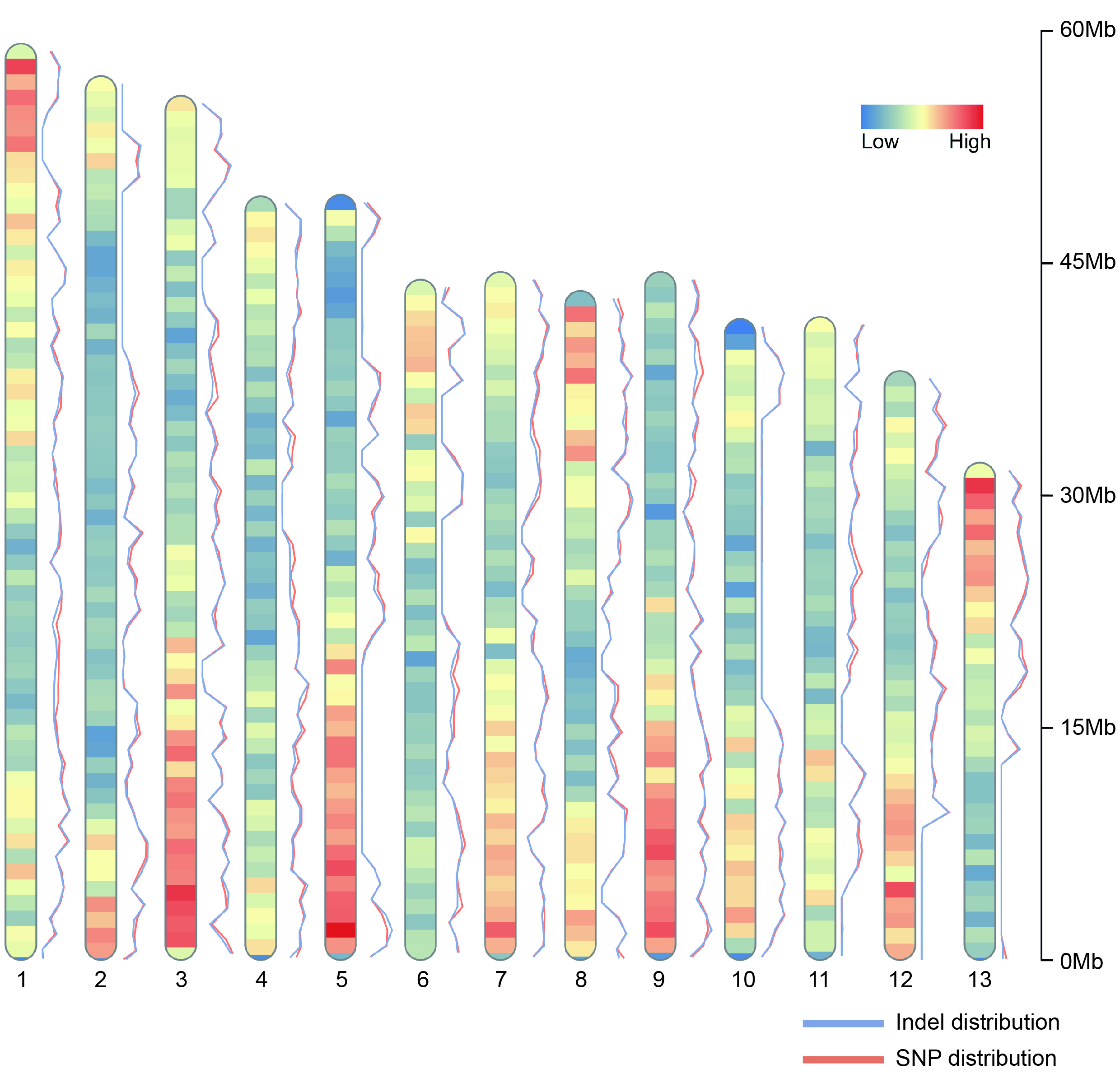


**Supplementary Figure 13.** SNP and Indel distribution between the two haplotypes.


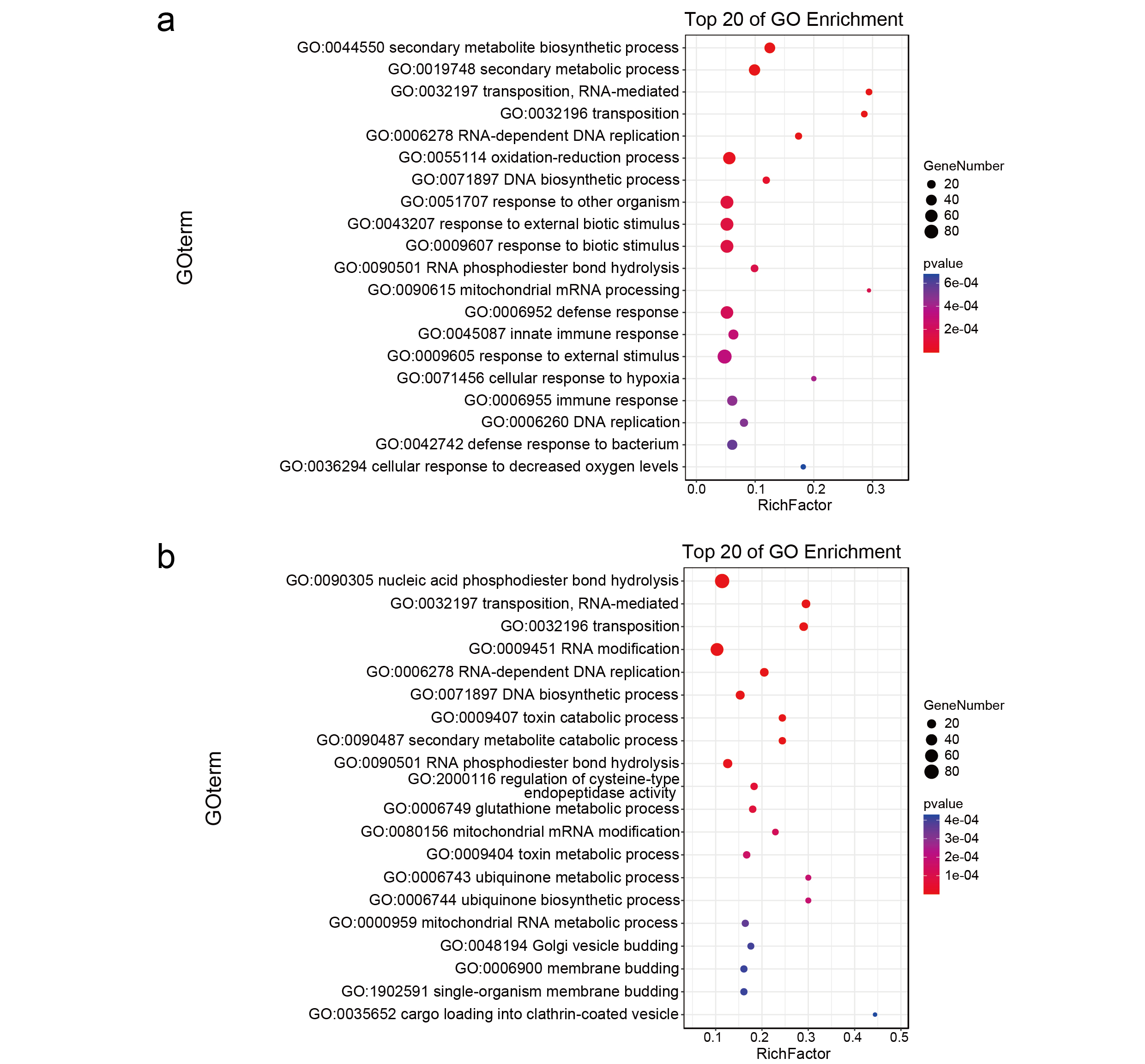


**Supplementary Figure 14.** Functional classification of GO annotations of haplotypes-specific genes (**a** Haplotype A-specific genes **b** Haplotype B-specific genes).


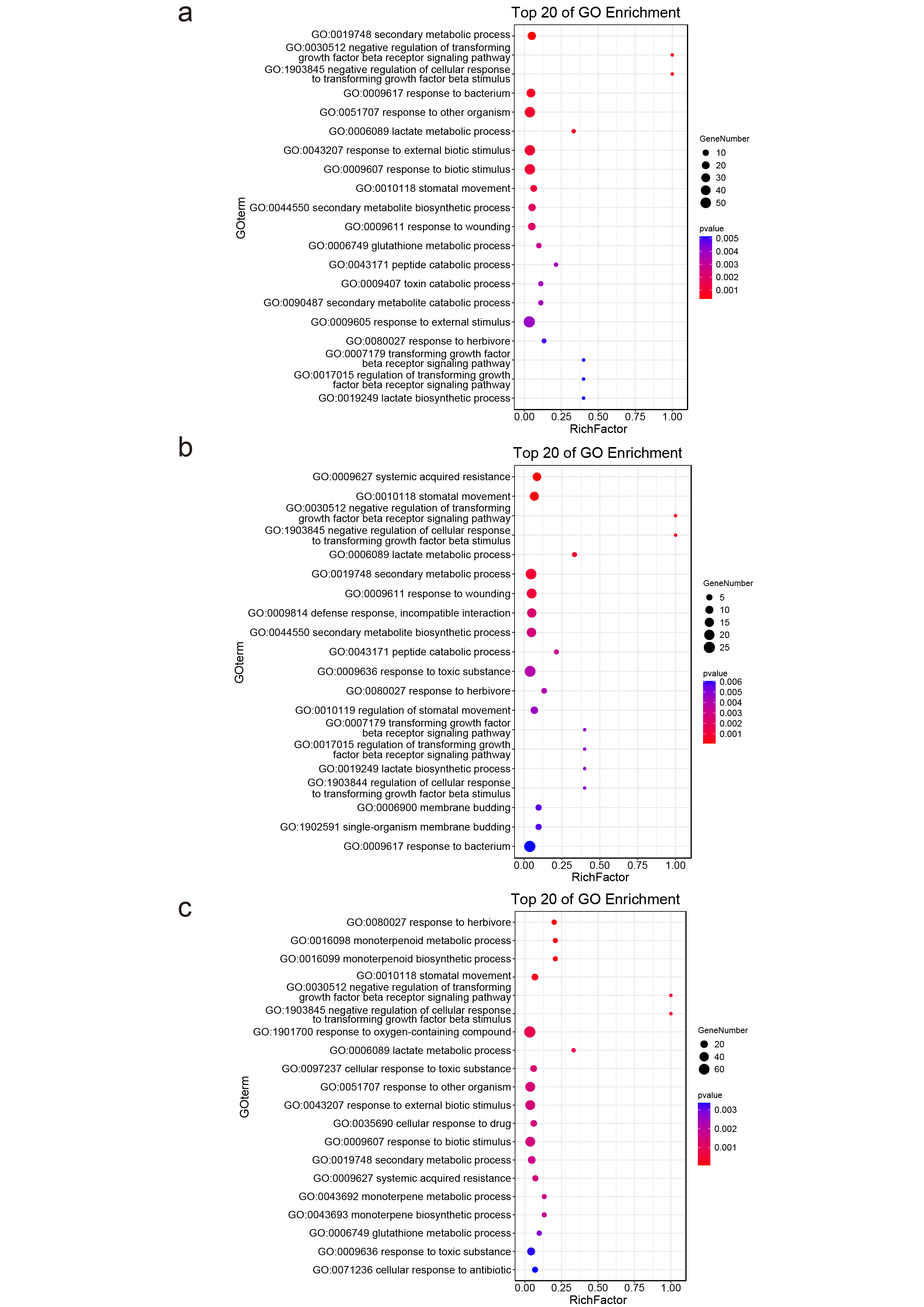


**Supplementary Figure 15** GO enrichments of DEGs from two haplotypes in TCL, NCL and R (**a** TCL **b** NCL **c** R)


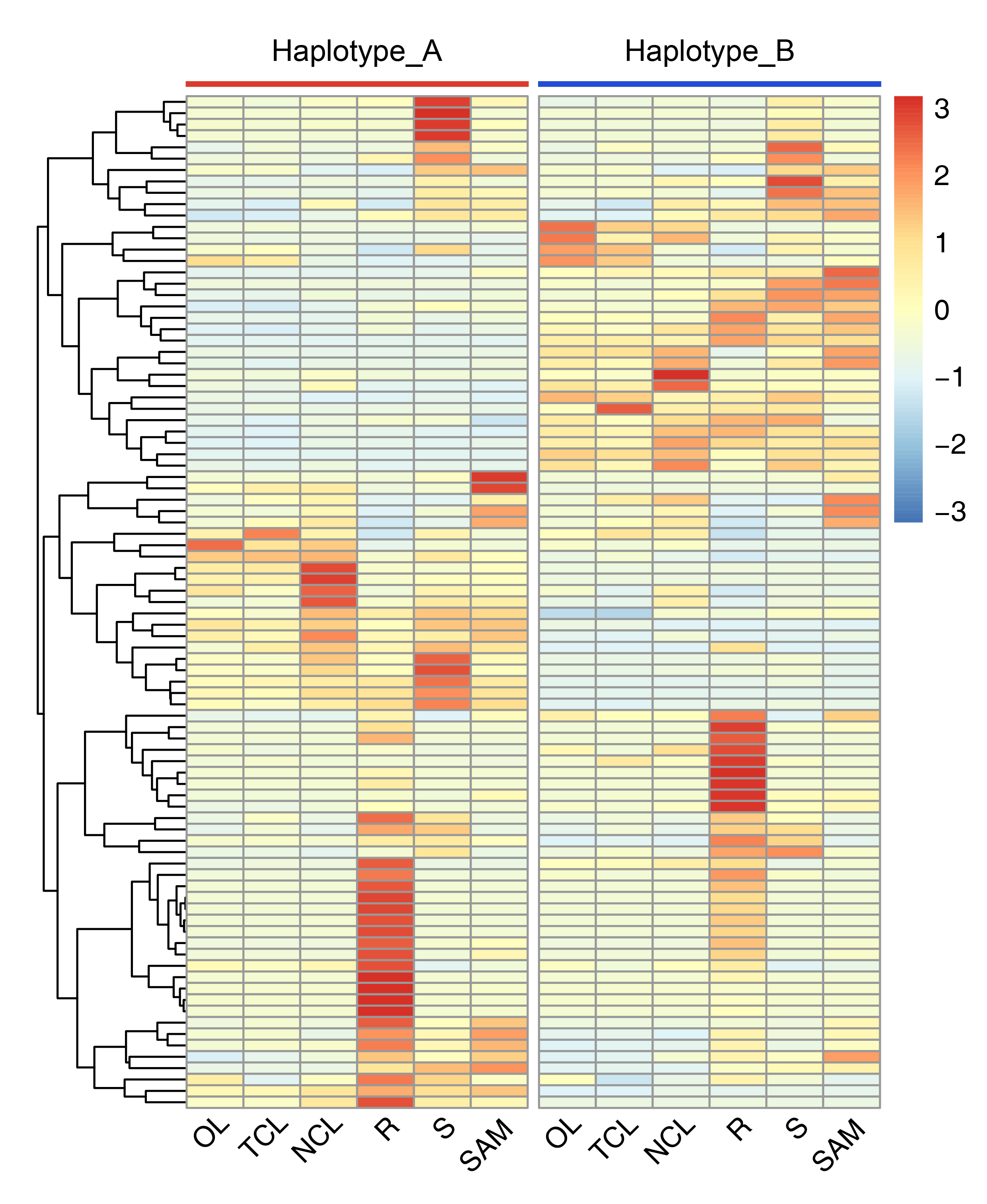


**Supplementary Figure 16.** Eighty-nine SV-related genes with different expression patterns in haplotype A and haplotype B


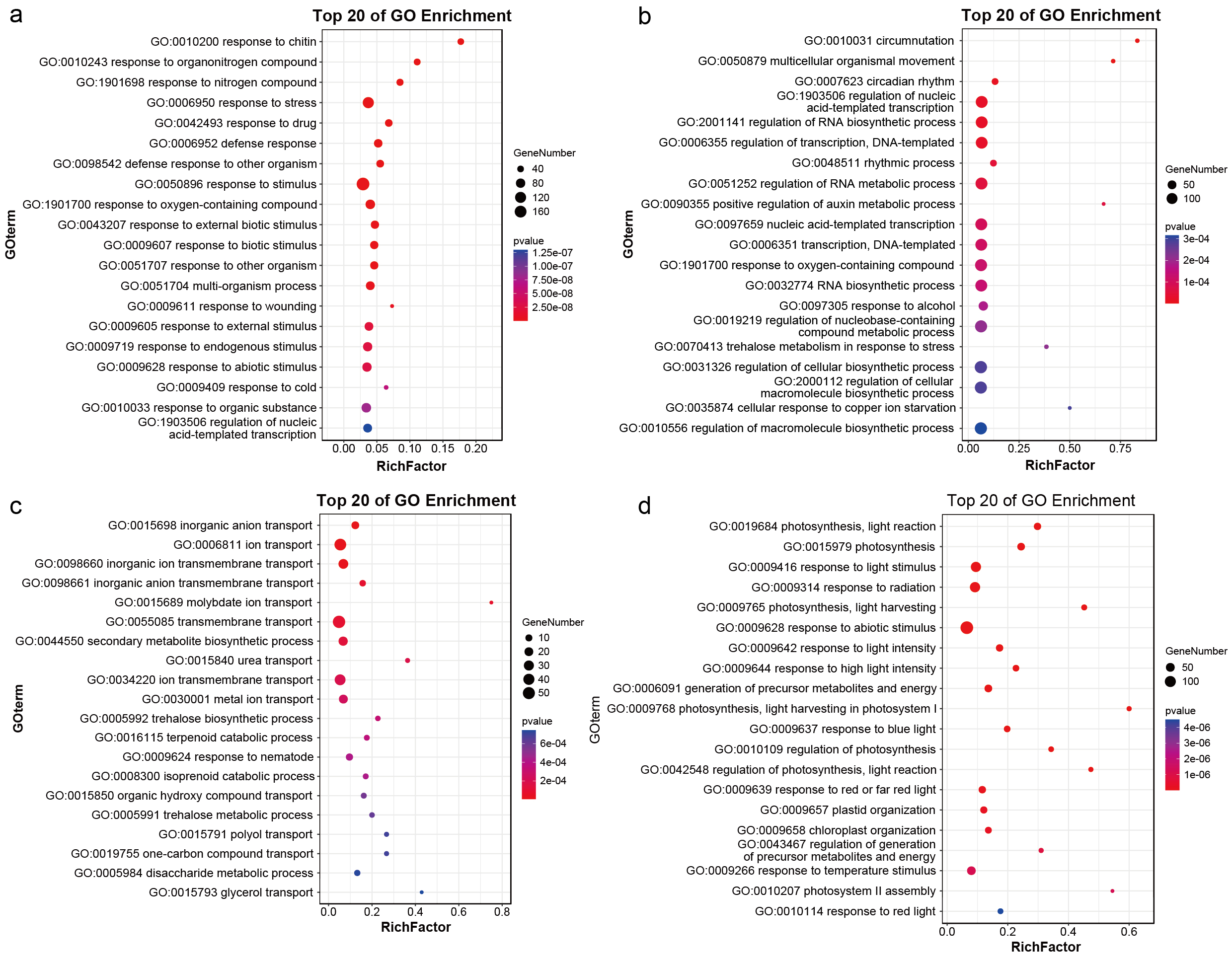


**Supplementary Figure 17.** GO enrichment analysis of DEGs in different expression clusters (biological process)

**a** GO terms of cluster 4 and cluster 6 in the biological process.

**b** GO terms of cluster1 and cluster5 in the biological process.

**c** GO terms of cluster 2 in the biological process.

**d** GO terms of cluster 3 in the biological process.


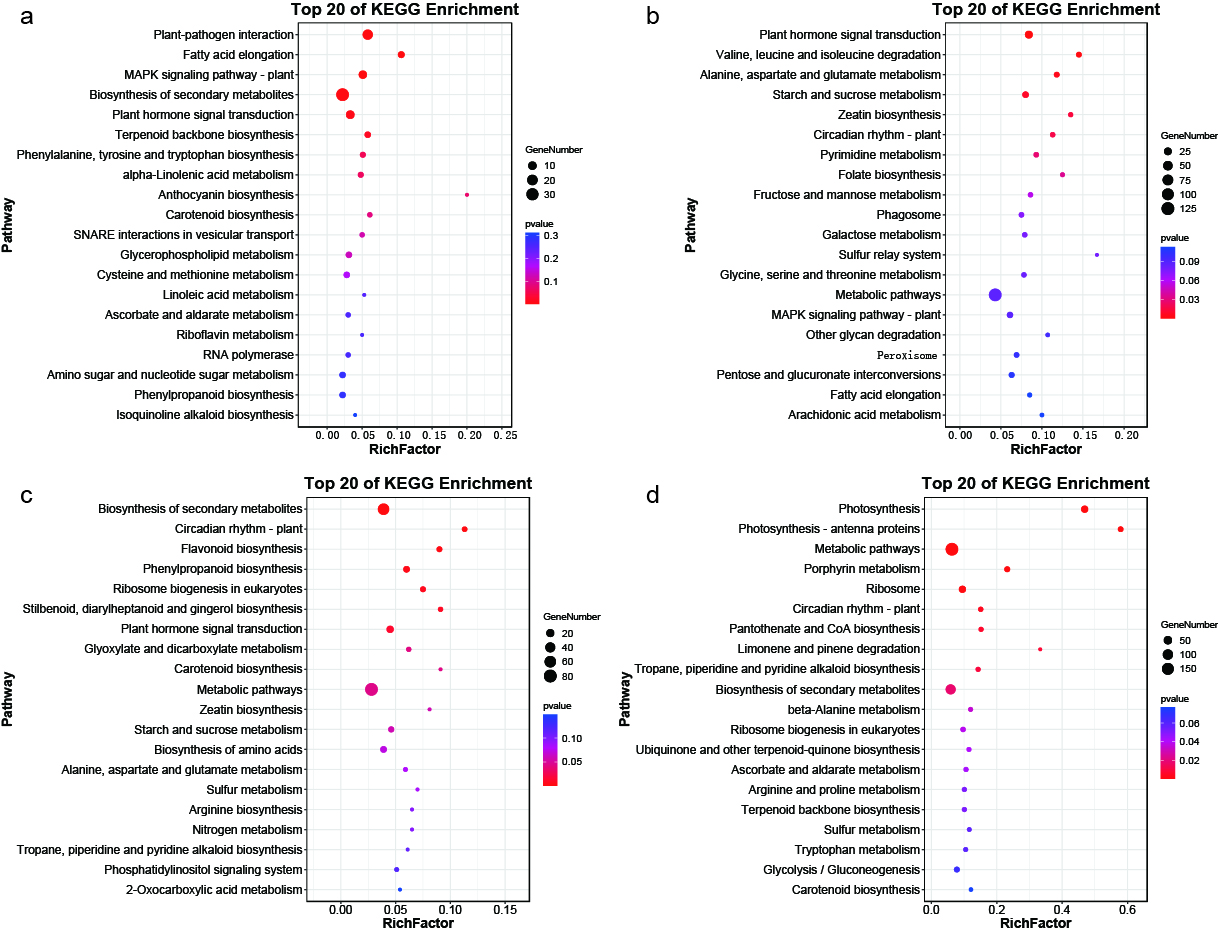


**Supplementary Figure 18.** KEGG enrichment analysis of DEGs in different expression clusters.

**a** KEGG terms of cluster 4 and cluster 6 in the biological process.

**b** KEGG terms of cluster1 and cluster5 in the biological process.

**c** KEGG terms of cluster 2 in the biological process.

**d** KEGG terms of cluster 3 in the biological process.


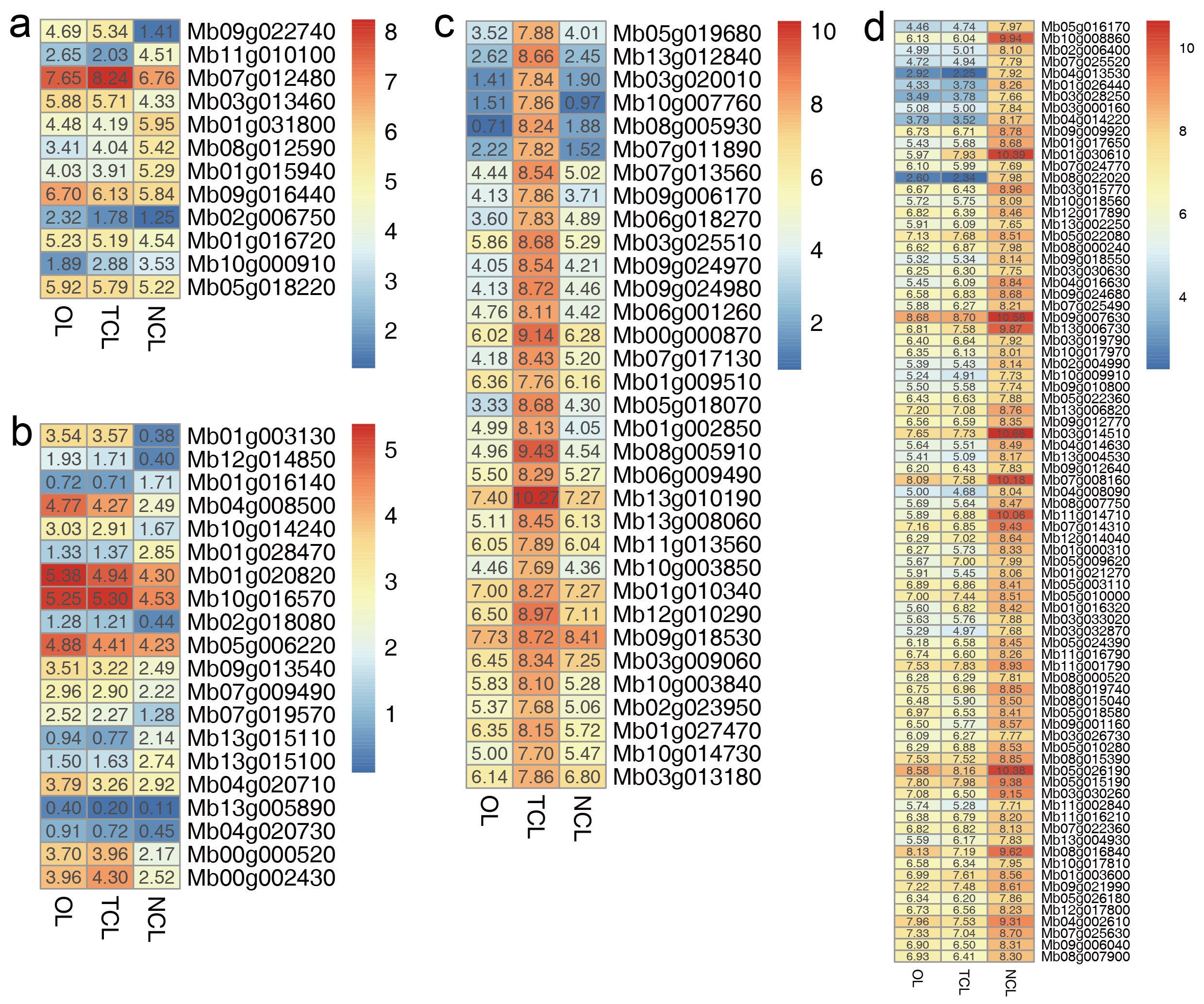


**Supplementary Figure 19.** The expression profile of candidate genes of Ca^2+^ related and ions related genes, and candidate genes with high expression involved in leaf movement. **a** The expression profile of candidate genes of Ca^2+^ related genes. **b** The expression profile of candidate genes of ions related genes. **c** The expression profile of candidate genes with high expression involved in [seismonastic](javascript:;) movement. **d** The expression profile of candidate genes with high expression involved in nyctinastic movement. The average FPKM of three biological replicates was used as the expression level, and they were normalized by Log2 (FPKM + 1).


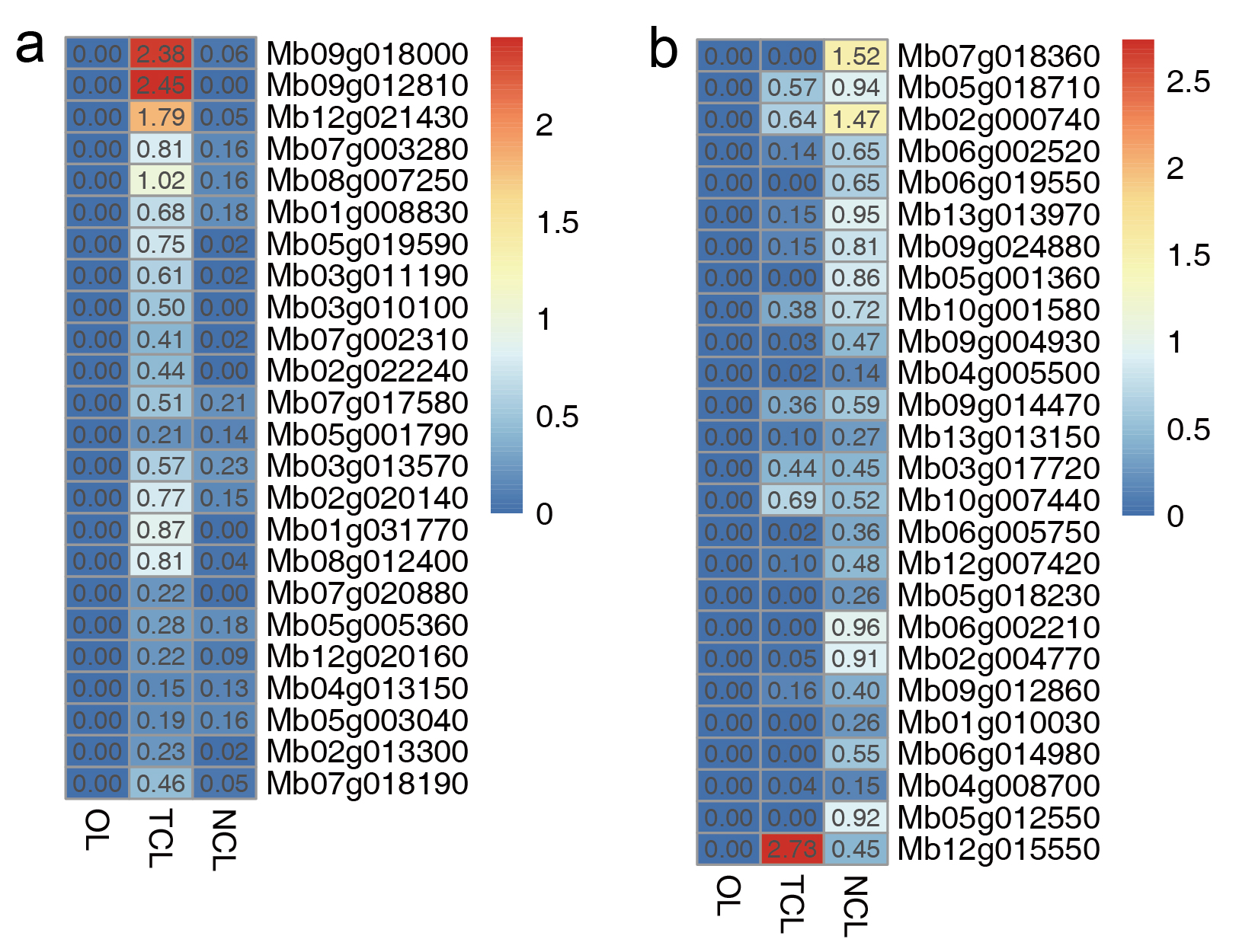


**Supplementary Figure 20.** The expression profile of candidate genes that were expressed specifically in different leaf states. **a** The expression profile of candidate genes that were expressed specifically in Touch-closed leaves **b** The expression profile of candidate genes that were expressed specifically in Night-closed-leaf. The average FPKM of three biological replicates was used as the expression level, and they were normalized by Log2 (FPKM + 1).


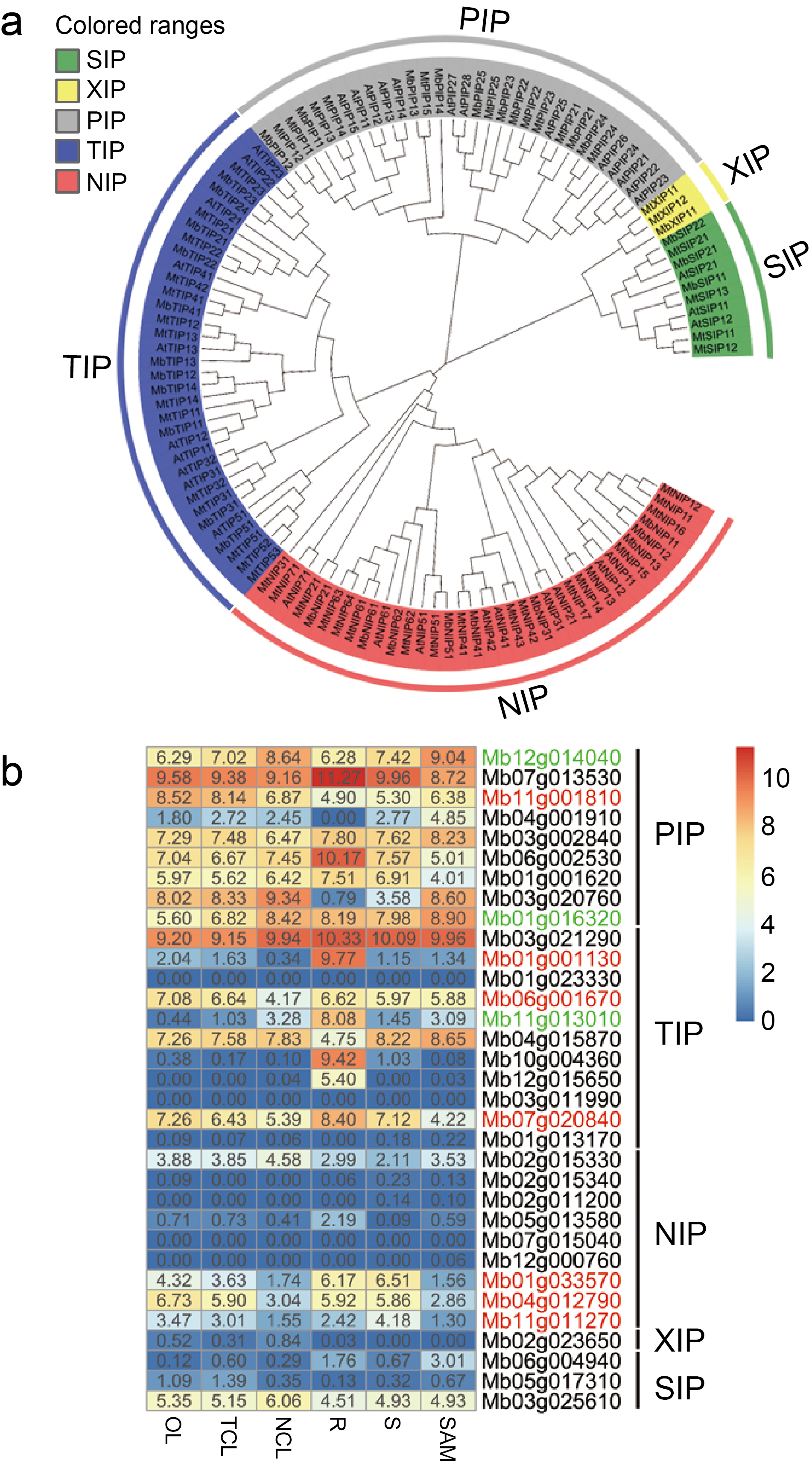


**Supplementary Figure 21.** Phylogenetic analysis and expression analysis of AQP genes.

**a** Phylogenetic tree of AQPs from *A. thaliana*, *M. truncatula* and *M. bimucronata.* The protein sequences were aligned by ClustalW and the phylogenetic tree was constructed by the neighbor-joining method (1000 bootstrap replicates) in the MEGAX software. The subfamilies are marked by a colorful background(blue for TIPs, gray for PIPs, red for NIPs, yellow for XIPs and green for SIPs.

**b** AQP genes expression profile in different tissues. Gene ID with color represents the DEGs found in the NCL.vs.OL and the red color represents the downregulated AQP genes, whereas the green color represents the upregulated AQP genes. The average FPKM of three biological replicates was used as the expression level, and they were normalized by Log2 (FPKM + 1).


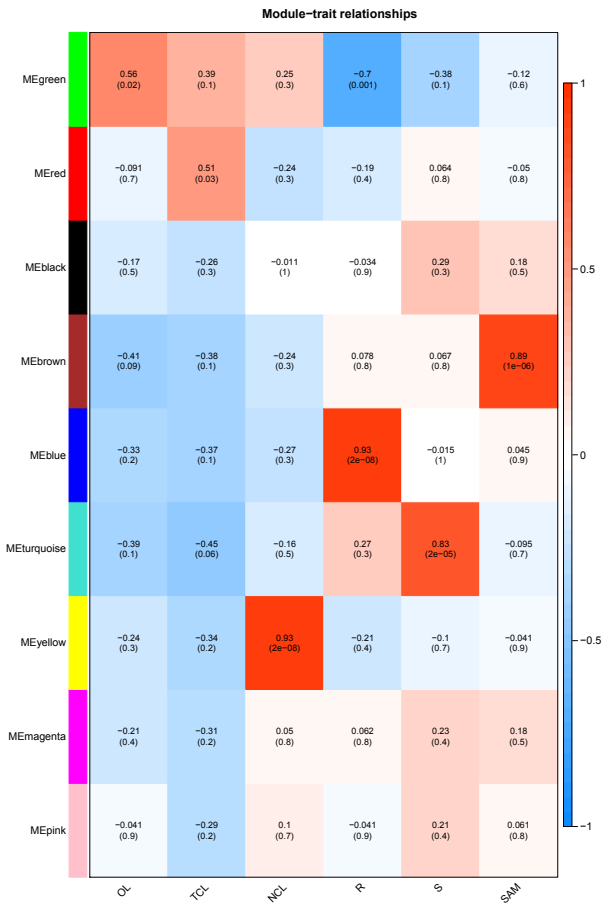


**Supplementary Figure 22.** Module-tissue association analysis.

Each row corresponds to a module and each column corresponds to a specific tissue. The correlation coefficient between a given module and tissue type is indicated by the color of the cell at the row-column intersection. Red and blue indicate

positive and negative correlations, respectively.


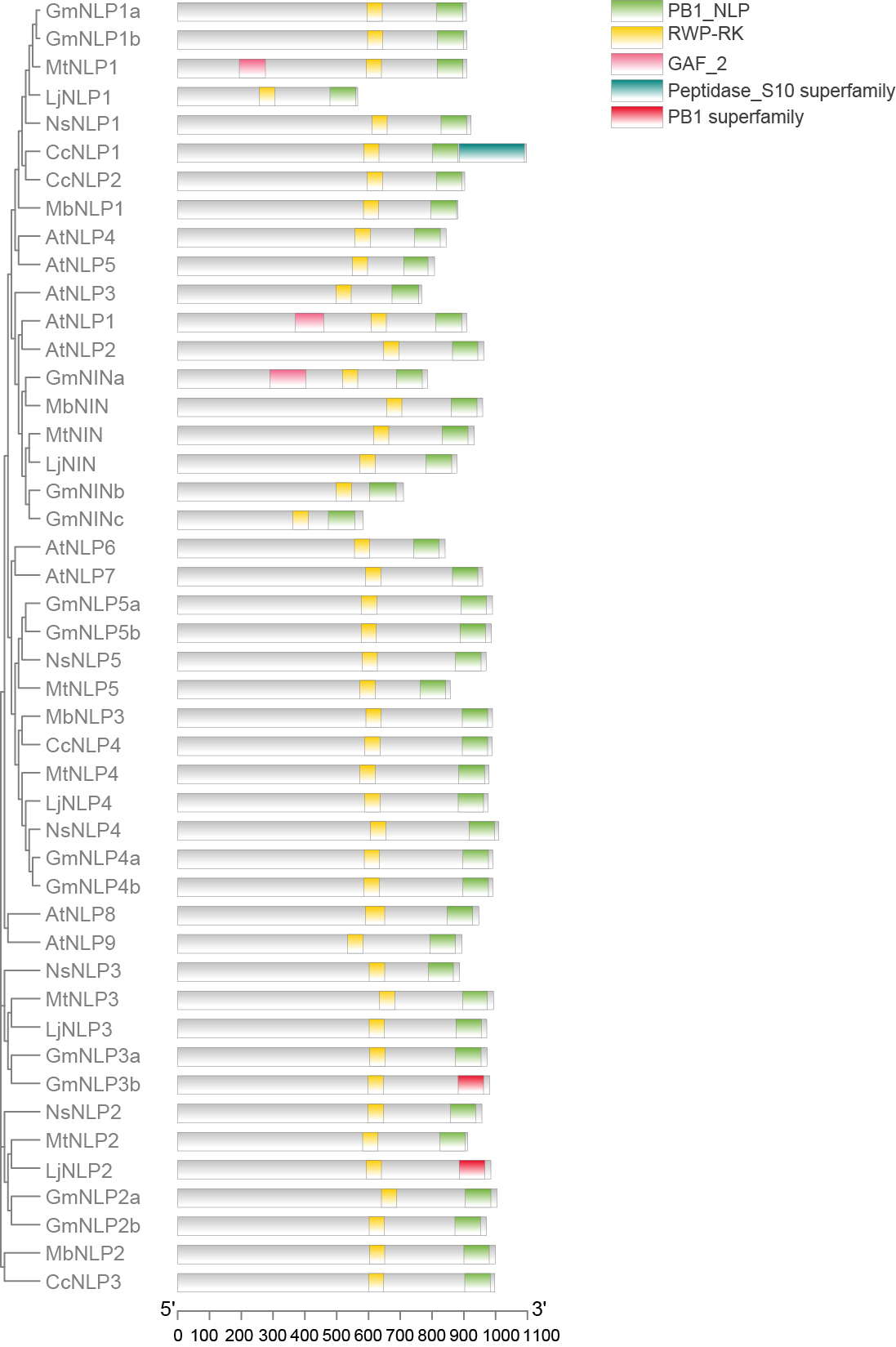


**Supplementary Figure 23.** Conserved domain analysis of NIN and NLP genes in seven species.


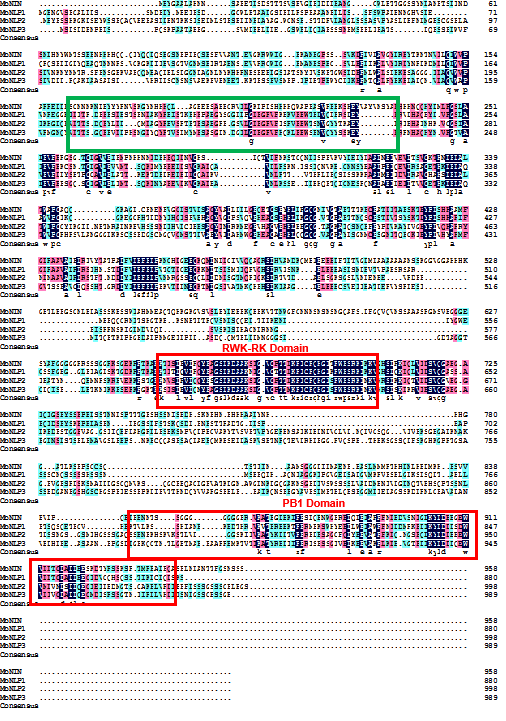


**Supplementary Figure 24.** Protein sequences of NIN and NLP genes [multiple sequence alignment](javascript:;) in *M. bimucronata.*

**Supplementary table**

**Supplementary Table 1** Genome size estimated by FCM

| **Sample** | **Internal reference** | **Genome** （Gb） | **Average (Gb)** | **Stdev** |
| --- | --- | --- | --- | --- |
| *Mimosa bimucronata* | *Zea mays* | 0.66 | 0.66 | 0.01 |
|  |  | 0.65 |  |  |
|  |  | 0.66 |  |  |

**Supplementary Table 2 Statistics of Illumina sequencing results**

| **Library** | **Insert size** | **Data (Gb)** | **Depth (×)** | **Q20 (%)** | **Q30 (%)** | **GC(%)** |
| --- | --- | --- | --- | --- | --- | --- |
| M1 | 350 bp | 27.14 | 41.12 | 97.83 | 93.73 | 34.30 |

***Supplementary Table 3 Genomic characteristics of M. bimucronata***

| Description |  |
| --- | --- |
| Genomesize | 654.60 Mb |
| heterozygosity | 1.01% |
| repetitiveness | 52.32% |
| ploidy | 2 |
| kmer | 17 |

**Supplementary Table 4** Statistics of PacBio sequencing and Illumina sequencing

| **Items** | **Sequencing platform** | |
| --- | --- | --- |
|  | **Pacbio HiFi** | **Illumina X10** |
| Total Number of reads | 1,020,303 | 181,340,050 |
| Total Number of sequenced Bases (Gb) | 17.34 | 27.14 |
| Mean reads length (bp) | 16,995 | 150 |
| N50 (bp) | 16,773 | 150 |
| Coverage (**×**) | ~26.27 | ~41.12 |

**Supplementary Table 5 Statistics of HiC sequencing**

| **Sample** | **Library** | **Read Pairs Number** | **Base Number** | **%≥Q30** |
| --- | --- | --- | --- | --- |
| 1. *bimucronata* | AL833-01H0001 | 198,353,890 | 59,267,283,088 | 93.71 |

**Supplementary** **Table 6** Statistics of Hi-C mapping

| Statistics of mapping | |
| --- | --- |
| Clean Paired-end Reads | 198353890 |
| Unmapped Paired-end Reads | 38169320 |
| Unmapped Paired-end Reads Rate (%) | 19.243 |
| Paired-end Reads with Singleton | 125784876 |
| Paired-end Reads with Singleton Rate (%) | 63.414 |
| Multi Mapped Paired-end Reads | 7827910 |
| Multi Mapped Ratio (%) | 3.946 |
| Unique Mapped Paired-end Reads | 26571784 |
| Unique Mapped Ratio (%) | 13.396 |
|  |  |
| Statistics of valid reads | |
| Dangling End Paired-end Reads | 13571901 |
| Dangling End Rate (%) | 51.076 |
| Self-Circle Paired-end Reads | 59510 |
| Self-Circle Rate (%) | 0.224 |
| Dumped Paired-end Reads | 11327 |
| Dumped Rate (%) | 0.04 |
| Interaction Paired-end Reads | 9228411 |
| Interaction Rate (%) | 34.73 |
| Valid Paired-end Reads | 7164319 |
| Valid Rate (%) | 77.633 |
| Cis_interaction | 3654670 |
| Trans_interaction | 3509649 |
| Cis_interaction/Trans_interaction | 1.041 |
| Cis_longRange | 3195060 |
| Cis_longRange Rate (%) | 87.424 |

**Supplementary Table** **7** Statistics of contig-level monoploid genome assembly

| **Items** | **Contig level assembly (HiFiasm)** | **After eliminating redundant sequences (Khaper)** | **Polished genome (Nextpolish)** |
| --- | --- | --- | --- |
|
| Assembly size (bp) | 672957931 | 648471567 | 648362449 |
| No. of contigs | 426 | 75 | 75 |
| Maximum length (bp) | 50387538 | 50102997 | 50079217 |
| N90 (bp) | 4567502 | 5625346 | 5623195 |
| N80 (bp) | 7548014 | 7867794 | 7863677 |
| N70 (bp) | 10210026 | 10866068 | 10860352 |
| N60 (bp) | 13746774 | 13960145 | 13952038 |
| N50 (bp) | 18254373 | 19040630 | 19036875 |
| Average length (bp) | 1579713 | 8646287 | 8642776 |

**Supplementary Table 8** Chromosome scale of the monoploid genome assembly

| **ChrID** | **No. Of anchored contigs** | **Length (bp)** |
| --- | --- | --- |
| Mbchr01 | 221 | 60083233 |
| Mbchr02 | 231 | 59872115 |
| Mbchr03 | 43 | 56480179 |
| Mbchr04 | 381 | 52050572 |
| Mbchr05 | 273 | 50175058 |
| Mbchr06 | 273 | 48410793 |
| Mbchr07 | 189 | 47359921 |
| Mbchr08 | 175 | 45723100 |
| Mbchr09 | 293 | 44751177 |
| Mbchr10 | 281 | 44251997 |
| Mbchr11 | 167 | 43072467 |
| Mbchr12 | 141 | 40074777 |
| Mbchr13 | 215 | 34611528 |
| Total number of contigs |  | 2883 |
| Total length of contigs |  | 648362449 |
| Total number of anchored contigs |  | 1449 |
| Total length of chromosome level assembly | | 626723017 |
| Number of unanchored contigs |  | 1434 |
| Length of unanchored contigs |  | 21639432 |
| Anchor rate (%) |  | 96.66 |

**Supplementary Table** **9** Assessment of genome consistency based on Illumina reads

| **Iterms** |  |
| --- | --- |
| Number of reads | 179028242 |
| Data size (Gb) | 27.14 |
| Mapping rate (%) | 96.47 |
| Genome Length (Mbp) | 648.36 |
| Mean Depth | 39.50 |
| Coverage Rate (%) | 99.99 |

**Supplementary Table 10** Assessment of genome assemblies based on RNA seq assembled transcripts

|  | **Number** | **Total Length (bp)** | **Accuracy(%)** | **Base covered by assembly(%)** | **Sequences covered by assembly(%)** | **With >90% sequence in same chromosome** | | **With >50% sequence in same chromosome** | |
| --- | --- | --- | --- | --- | --- | --- | --- | --- | --- |
|  |  |  |  |  |  | **Number** | **Percent(%)** | **Number** | **Percent(%)** |
| RNA assembled | 161207 | 405630583 | 99.99% | 100.00% | 99.99% | 161206 | 99.99% | 161206 | 99.99% |
| transcripts |  |  |  |  |  |  |  |  |  |

**Supplementary Table 11 BUSCO completeness analysis of the monoploid genome assembly and annotation**

| **Descripation** | **Genome** | | **Annotation** | |
| --- | --- | --- | --- | --- |
|  | **Number** | **Percentage(%)** | **Number** | **Percentage(%)** |
| Complete BUSCOs (C) | 1592 | 98.70% | 1589 | 98.40% |
| Complete and single copy BUSCOs (S) | 1523 | 94.40% | 1514 | 93.80% |
| Complete and duplicated BUSCOs (D) | 69 | 4.30% | 75 | 4.60% |
| Fragmented BUSCOs (F) | 6 | 0.40% | 17 | 1.10% |
| Missing BUSCOs (M) | 16 | 0.90% | 8 | 0.50% |
| Total BUSCO groups searched | 1614 | 100.00% | 1614 | 100.00% |

**Supplementary Table 12** BUSCO analysis of annotation completeness in the *M. bimucronata* haplotype-resolved genome

| **Descripation** | **Haplotype A** | | | | **Haplotype B** | | | |
| --- | --- | --- | --- | --- | --- | --- | --- | --- |
|  | **Genome** | | **Annotation** | | **Genome** | | **Annotation** | |
|  | **Number** | **Percentage**  **(%)** | **Number** | **Percentage**  **(%)** | **Number** | **Percentage**  **(%)** | **Number** | **Percentage (%)** |
| Complete BUSCOs (C) | 1573 | 97.4 | 1546 | 95.8 | 1588 | 98.4 | 1574 | 97.5 |
| Complete and single copy BUSCOs (S) | 1508 | 93.4 | 1486 | 92.1 | 1518 | 94.1 | 1499 | 92.9 |
| Complete and duplicated BUSCOs (D) | 65 | 4 | 60 | 3.7 | 70 | 4.3 | 75 | 4.6 |
| Fragmented BUSCOs (F) | 6 | 0.4 | 30 | 1.9 | 8 | 0.5 | 25 | 1.5 |
| Missing BUSCOs (M) | 35 | 2.2 | 38 | 2.3 | 18 | 1.1 | 15 | 1 |
| Total BUSCO groups searched | 1614 | 100 | 1614 | 100 | 1614 | 100 | 1614 | 100 |

**Supplementary Table 13** Statistics of gene function annotation of the *M. bimucronata* monoploid genome

| **Database** | **Number** | **Percentage(%)** |
| --- | --- | --- |
| Total | 32146 | 100 |
| EggNOGmapper | 28912 | 86.31 |
| KEGG | 12433 | 35.27 |
| Swisspro | 25032 | 76.38 |
| KOG | 29055 | 87.32 |
| NR | 31128 | 96.83 |
| Annotated | 31137 | 96.86 |
| Unannotated | 1009 | 3.14 |

**Supplementary Table 14** Number of noncoding RNAs in the *M. bimucronata* monoploid genome

| **Type** | **Number** |
| --- | --- |
| tRNA | 165 |
| miRNA | 99 |
| rRNA | 39 |
| snRNA | 336 |

**Supplementary Table 15** Statistics of repeat sequences in the *M. bimucronata* monoploid genome

|  | **Length (Mb)** | **% of genome** |
| --- | --- | --- |
| **Total repeat fraction** | 369.85 | 57.04 |
| **Class I: Retroelement** | 230.78 | 35.59 |
| **LTR Retrotransposon** | 149.06 | 22.99 |
| Ty1/Copia | 43.47 | 6.7 |
| Ty3/Gypsy | 40.72 | 6.28 |
| Other | 64.88 | 10.01 |
| **Non LTR Retrotransposon** | 54.46 | 8.4 |
| LINE | 52.05 | 8.03 |
| SINE | 2.41 | 0.37 |
| **Unclassified retroelement** | 27.26 | 4.2 |
| **Class II: DNA Transposon** | 103.61 | 15.98 |
| **TIR** |  |  |
| CMC | 30.32 | 4.68 |
| hAT | 2.35 | 0.36 |
| Mutator | 4.12 | 0.64 |
| Tc1/Mariner | 0 | 0 |
| PIF/Harbinger | 0.49 | 0.08 |
| Other | 66.32 | 10.23 |
| **Helitron** | 4.73 | 0.73 |
| **Tandem Repeats** | 33.85 | 5.22 |
| **Unkown** | 14.68 | 2.26 |

**Supplementary Table 16** Statistics of repeat sequences between the two haplotypes in the *M. bimucronata* genome

| **Descriptions** | **HaplotypeA** | | **HaplotypeB** | |
| --- | --- | --- | --- | --- |
|  | **Length (M)** | **% of genome** | **Length (M)** | **% of genome** |
| **Total repeat fraction** | 366.22 | 58.27 | 383.28 | 58.57 |
| **Class I: Retroelement** | 221.27 | 35.21 | 228.62 | 34.94 |
| **LTR Retrotransposon** | 135.32 | 21.53 | 147.14 | 22.49 |
| Ty1/Copia | 41.1 | 6.54 | 43.19 | 6.6 |
| Ty3/Gypsy | 33.22 | 5.29 | 37.21 | 5.69 |
| Other | 61 | 9.71 | 66.75 | 10.2 |
| **Non LTR Retrotransposon** | 59.56 | 9.48 | 54.24 | 8.29 |
| LINE | 52.81 | 8.4 | 50.98 | 7.79 |
| SINE | 6.75 | 1.07 | 3.26 | 0.5 |
| **Unclassified retroelement** | 26.38 | 4.2 | 27.24 | 4.16 |
| **Class II: DNA Transposon** | 106.48 | 16.94 | 105.59 | 16.14 |
| **TIR** |  |  |  |  |
| CMC | 29.42 | 4.68 | 29.91 | 4.57 |
| hAT | 3.94 | 0.63 | 2.37 | 0.36 |
| Mutator | 2.71 | 0.43 | 3.59 | 0.55 |
| Tc1/Mariner | 0 | 0 | 0.04 | 0.01 |
| PIF/Harbinger | 0.89 | 0.14 | 0.54 | 0.08 |
| Other | 69.52 | 11.06 | 69.1 | 10.56 |
| **Helitron** | 9.82 | 1.56 | 14.78 | 2.26 |
| **Tandem Repeats** | 29.91 | 4.76 | 31.78 | 4.86 |
| **Unkown** | 15.28 | 2.43 | 14.76 | 2.26 |

**Supplementary Table 17** Sources of seven species used in the phylogenetic analysis

| **Species** | **Source** |
| --- | --- |
| *Arabidopsis thaliana* | <https://phytozome-next.jgi.doe.gov/info/Athaliana_TAIR10> |
| *Oryza sativa* | <https://phytozome-next.jgi.doe.gov/info/Osativa_v7_0> |
| *Carica papaya* | <https://phytozome-next.jgi.doe.gov/info/Cpapaya_ASGPBv0_4> |
| *Dalbergia odorifera* | http://gigadb.org/dataset/100760 |
| *Faidherbia albida* | <https://bioinformatics.psb.ugent.be/gdb/aocc/faial/> |
| *Medicago truncatula* | <https://phytozome-next.jgi.doe.gov/info/Mtruncatula_Mt4_0v1> |
| *Senna tora* | <http://nabic.rda.go.kr/Species/Senna_tora2> |

**Supplementary Table 18** Overall orthogroups in 8 species

| Descriptions |  |
| --- | --- |
| Number of species | 8 |
| Number of genes | 310013 |
| Number of genes in orthogroups | 220897 |
| Number of unassigned genes | 89116 |
| Percentage of genes in orthogroups | 71.3 |
| Percentage of unassigned genes | 28.7 |
| Number of orthogroups | 17824 |
| Number of species-specific orthogroups | 707 |
| Number of genes in species-specific orthogroups | 4631 |
| Percentage of genes in species-specific orthogroups | 1.5 |
| Mean orthogroup size | 12.4 |
| Median orthogroup size | 10 |
| G50 (assigned genes) | 14 |
| G50 (all genes) | 11 |
| O50 (assigned genes) | 4413 |
| O50 (all genes) | 8011 |
| Number of orthogroups with all species present | 9332 |
| Number of single-copy orthogroups | 920 |

**Supplementary Table 19** Statistics of orthogroups in PerSpecies

| Descriptions | At | Cp | Do | Fa | Mb | Mt | Os | St |
| --- | --- | --- | --- | --- | --- | --- | --- | --- |
| Number of genes | 35386 | 23181 | 30310 | 28979 | 32146 | 62319 | 52424 | 45268 |
| Number of genes in orthogroups | 28485 | 18324 | 24612 | 24410 | 28822 | 41645 | 27966 | 26633 |
| Number of unassigned genes | 6901 | 4857 | 5698 | 4569 | 3324 | 20674 | 24458 | 18635 |
| Percentage of genes in orthogroups | 80.5 | 79 | 81.2 | 84.2 | 89.7 | 66.8 | 53.3 | 58.8 |
| Percentage of unassigned genes | 19.5 | 21 | 18.8 | 15.8 | 10.3 | 33.2 | 46.7 | 41.2 |
| Number of orthogroups containing species | 13052 | 13283 | 14463 | 14669 | 14916 | 14917 | 12524 | 14112 |
| Percentage of orthogroups containing species | 73.2 | 74.5 | 81.1 | 82.3 | 83.7 | 83.7 | 70.3 | 79.2 |
| Number of species-specific orthogroups | 107 | 35 | 18 | 13 | 28 | 121 | 274 | 111 |
| Number of genes in species-specific orthogroups | 662 | 245 | 67 | 39 | 178 | 1022 | 1561 | 857 |
| Percentage of genes in species-specific orthogroups | 1.9 | 1.1 | 0.2 | 0.1 | 0.6 | 1.6 | 3 | 1.9 |

**Supplementary Table 21 Number of expanded and contracted gene families and rapidly evolving families**

| **Node** | **Expansions** | **Contractions** | **Rapidly evolving families** |
| --- | --- | --- | --- |
| St<8> | 1575 | 3870 | 225 |
| Fa<6> | 1037 | 1940 | 126 |
| Mt<10> | 6337 | 1375 | 337 |
| <3> | 184 | 2203 | 5 |
| At<0> | 4801 | 2286 | 72 |
| Os<14> | 4546 | 6991 | 16 |
| Do<12> | 2345 | 2757 | 67 |
| <1> | 39 | 3283 | 3 |
| <7> | 508 | 1645 | 77 |
| <5> | 990 | 1365 | 127 |
| <9> | 1118 | 753 | 48 |
| Mb<4> | 1817 | 1078 | 222 |
| Cp<2> | 691 | 4528 | 39 |
| <11> | 846 | 1576 | 40 |

**Supplementary Table 22** Statistics of genetic variation between the two haplotypes in the *M. bimucronata* genome

| **ChrID** | **No. of SNPs** | **Small Indels (<50 bp)** | | **Large Indels (>50 bp)** | |
| --- | --- | --- | --- | --- | --- |
|  |  | **No. of insertions** | **No. of deletions** | **No. of insertions** | **No. of deletions** |
| Chr01 | 488,188 | 13,841 | 13,590 | 620 | 512 |
| Chr02 | 283,457 | 8,012 | 8,046 | 333 | 273 |
| Chr03 | 430,439 | 12,359 | 12,153 | 312 | 409 |
| Chr04 | 364,417 | 10,333 | 10,216 | 456 | 322 |
| Chr05 | 199,258 | 5,709 | 5,552 | 246 | 236 |
| Chr06 | 248,081 | 6,952 | 7,002 | 293 | 255 |
| Chr07 | 363,336 | 10,336 | 9,946 | 476 | 367 |
| Chr08 | 368,721 | 10,458 | 10,217 | 479 | 361 |
| Chr09 | 380,113 | 10,323 | 10,444 | 500 | 377 |
| Chr10 | 199,066 | 5,969 | 5,722 | 226 | 192 |
| Chr11 | 268,886 | 7,278 | 7,298 | 311 | 269 |
| Chr12 | 204,013 | 5,736 | 5,658 | 271 | 199 |
| Chr13 | 164,382 | 4,791 | 4,760 | 197 | 185 |
| Total | 3,962,357 | 112,097 | 110,604 | 4,720 | 3,957 |

**Supplementary Table 23** Experimental validation of 20 large indels

| **Type** | **Mark_in_Gel** | **Ref**  **chr** | **Ref**  **start** | **Ref**  **end** | **Query**  **chr** | **Query**  **start** | **Query**  **end** | **Indel**  **length (bp)** | **Forward Primer** | **Reverse Primer** | **product**  **length (+)** | **product**  **length (-)** |
| --- | --- | --- | --- | --- | --- | --- | --- | --- | --- | --- | --- | --- |
| deletion | D1 | HapA_Chr1 | 2,635,655 | 2,636,653 | HapB_Chr1 | 2,472,055 | 2,472,813 | 240 | ACCATCCTTGCGGGTAAAGG | GAAAGAGCCAAGGACGGACA | 521 | 281 |
|  | D2 | HapA_Chr1 | 13,881,984 | 13,882,982 | HapB_Chr1 | 13,791,066 | 13,791,824 | 240 | TCAGCACTGAAGCCGACAAT | TTGAGGCAGTTCCACGGATTC | 565 | 325 |
|  | D3 | HapA_Chr1 | 13,998,595 | 13,999,594 | HapB_Chr1 | 13,897,750 | 13,898,508 | 241 | GGAGACCTTGGAGCTCAATT | CACATATCCGCATTCTCAAACCA | 587 | 346 |
|  | D4 | HapA_Chr1 | 15,980,720 | 15,981,718 | HapB_Chr1 | 15,984,153 | 15,984,930 | 221 | CCTGGACCCATTGCCCAAAT | TCAATCGATTGTCCAAGAGCCA | 580 | 359 |
|  | D5 | HapA_Chr1 | 20,547,347 | 20,548,346 | HapB_Chr1 | 21,175,506 | 21,176,245 | 266 | TGAAGTGTGTGAGAAGGGAAC | GGAGGAGAGATATGTACTTGTCACA | 573 | 307 |
|  | D6 | HapA_Chr1 | 22,146,776 | 22,147,775 | HapB_Chr1 | 22,766,259 | 22,766,861 | 397 | TCGTTTGATTGGACCGGTGA | TGACAGGAAATGACATGGTAGAGT | 582 | 185 |
|  | D7 | HapA_Chr1 | 47,605,526 | 47,606,524 | HapB_Chr1 | 48,064,553 | 48,065,325 | 226 | CCATCATCAACTTGTTCGGCA | TAGTTGTTGCCGAGGTGTGG | 566 | 340 |
|  | D8 | HapA_Chr1 | 49,601,924 | 49,602,923 | HapB_Chr1 | 51,147,934 | 51,148,932 | 239 | TCGTTGTAGGTTGGAGCTCAC | GACAGAAACCAAAACGGGCTC | 600 | 361 |
|  | D9 | HapA_Chr1 | 51,147,934 | 51,148,932 | HapB_Chr1 | 51,727,771 | 51,728,441 | 328 | GCGGTTGCCTGCTACTAATT | CACTTTCGCAGGCAAACACA | 555 | 227 |
|  | D10 | HapA_Chr1 | 53,916,043 | 53,917,041 | HapB_Chr1 | 54,758,957 | 54,759,747 | 208 | GGGTGAAAATTGGGCCCGTA | AGACACTCAGTGCCCCTTTG | 504 | 296 |
| insertion | I1 | HapA_Chr1 | 10,357,913 | 10,358,912 | HapB_Chr1 | 10,344,346 | 10,345,610 | 266 | ACTACCTTTGGTCTGTCAGCT | GGAGAAGCCGTGGGGTTTTA | 532 | 266 |
|  | I2 | HapA_Chr1 | 10,973,163 | 10,974,162 | HapB_Chr1 | 10,954,035 | 10,955,272 | 239 | GCATCCCAACTGCCTCAGAA | TGGGCGGAAACTGATGAAGG | 585 | 346 |
|  | I3 | HapA_Chr1 | 17,728,529 | 17,729,528 | HapB_Chr1 | 17,794,345 | 17,795,720 | 377 | AGTCTTCGGGTGGCAAAAGA | GAAGAAGCTGTGGGTGTCCA | 926 | 549 |
|  | I4 | HapA_Chr1 | 25,376,363 | 25,377,362 | HapB_Chr1 | 25,818,279 | 25,819,543 | 266 | CGAGGAGCTTTTTGGCAGTG | AGCACACACATGACAAGACTGA | 591 | 325 |
|  | I5 | HapA_Chr1 | 33,086,476 | 33,087,475 | HapB_Chr1 | 33,193,620 | 33,194,940 | 322 | AAGCAAAGTCTACGGTGGGG | ACCATCAAGCACGTCGGAAT | 546 | 224 |
|  | I6 | HapA_Chr1 | 37,852,576 | 37,853,575 | HapB_Chr1 | 38,282,757 | 38,284,018 | 263 | TGCACCTCTCCTCCTGATTT | TCCGAATTCATATTGTTGTCATGAGT | 502 | 239 |
|  | I7 | HapA_Chr1 | 41,320,349 | 41,321,348 | HapB_Chr1 | 41,803,693 | 41,805,096 | 405 | GCTTTGCACCTTCCTTCTTCA | CTGTTAGGACAACCTCTCCC | 539 | 134 |
|  | I8 | HapA_Chr1 | 44,304,329 | 44,305,327 | HapB_Chr1 | 44,822,655 | 44,823,937 | 290 | TGCAAAGCCCAATGTCACAC | GAAATCCTTTGGGTCTTGGCT | 526 | 236 |
|  | I9 | HapA_Chr1 | 53,284,449 | 53,285,447 | HapB_Chr1 | 54,125,797 | 54,127,083 | 306 | TTCAGTAACTGCACTGAGA | GGCATGCAAGAGAGGACTGA | 640 | 334 |
|  | I10 | HapA_Chr1 | 54,477,631 | 54,478,630 | HapB_Chr1 | 55,334,704 | 55,336,101 | 399 | TGGGCCCTCCTTCTAAATGT | CATCAACTGTCGGCGTCTTT | 604 | 205 |

**Supplementary Table 24** Number of genes showing differential expression toward haplotype A (i.e., A>B) or haplotype B (A<B)

| **ChrID** | **OL** | | **TCL** | | **NCL** | | | **R** | | **S** | | **T** | |
| --- | --- | --- | --- | --- | --- | --- | --- | --- | --- | --- | --- | --- | --- |
|  | **A>B** | **A<B** | **A>B** | **A<B** | **A>B** | **A<B** | **A>B** | | **A<B** | **A>B** | **A<B** | **A>B** | **A<B** |
| Chr01 | 34 | 52 | 39 | 52 | 31 | 54 | | 43 | 46 | 40 | 48 | 39 | 43 |
| Chr02 | 28 | 35 | 28 | 34 | 24 | 39 | | 25 | 35 | 27 | 34 | 27 | 37 |
| Chr03 | 21 | 29 | 23 | 38 | 23 | 30 | | 23 | 32 | 23 | 33 | 21 | 35 |
| Chr04 | 20 | 22 | 27 | 19 | 24 | 19 | | 26 | 23 | 26 | 24 | 25 | 23 |
| Chr05 | 16 | 26 | 18 | 25 | 17 | 24 | | 17 | 24 | 18 | 24 | 6 | 23 |
| Chr06 | 5 | 14 | 7 | 14 | 7 | 11 | | 7 | 15 | 8 | 6 | 6 | 14 |
| Chr07 | 17 | 30 | 17 | 31 | 20 | 27 | | 16 | 31 | 18 | 31 | 22 | 29 |
| Chr08 | 34 | 22 | 36 | 24 | 34 | 19 | | 35 | 20 | 36 | 23 | 39 | 22 |
| Chr09 | 28 | 23 | 30 | 26 | 26 | 28 | | 33 | 20 | 32 | 25 | 30 | 23 |
| Chr10 | 7 | 15 | 8 | 12 | 12 | 14 | | 11 | 15 | 10 | 16 | 9 | 13 |
| Chr11 | 19 | 12 | 11 | 13 | 12 | 10 | | 18 | 12 | 17 | 13 | 15 | 12 |
| Chr12 | 12 | 19 | 14 | 20 | 13 | 17 | | 10 | 26 | 12 | 19 | 12 | 21 |
| Chr13 | 9 | 15 | 7 | 15 | 8 | 14 | | 12 | 13 | 11 | 15 | 7 | 13 |

**Supplementary Table 26** Transcriptome data from 6 tissues of *M. bimucronata*

| **Tissue** | **Samples** | **RAW_READS** | **RAW_BASES** | **READ_LENGTH** | **RawQ30** |
| --- | --- | --- | --- | --- | --- |
| Root | R1 | 31,351,672 | 9,405,501,600 | 150;150 | 91.29;88.61 |
|  | R2 | 23,723,842 | 7,117,152,600 | 150;150 | 92.75;91.23 |
|  | R3 | 20,435,464 | 6,130,639,200 | 150;150 | 92.89;90.03 |
| Stem | S1 | 23,324,942 | 6,997,482,600 | 150;150 | 92.64;89.98 |
|  | S2 | 25,400,821 | 7,620,246,300 | 150;150 | 92.93;90.99 |
|  | S3 | 26,954,312 | 8,086,293,600 | 150;150 | 92.72;91.26 |
| Stem apical meristem | SAM1 | 26,122,142 | 7,836,642,600 | 150;150 | 92.16;89.34 |
|  | SAM2 | 20,983,216 | 6,294,964,800 | 150;150 | 93.06;90.97 |
|  | SAM3 | 27,120,520 | 8,136,156,000 | 150;150 | 92.80;90.75 |
| Open leaf | OL1 | 25,186,926 | 7,556,077,800 | 150;150 | 92.79;90.58 |
|  | OL2 | 24,114,192 | 7,234,257,600 | 150;150 | 93.21;90.98 |
|  | OL3 | 24,643,663 | 7,393,098,900 | 150;150 | 93.08;91.16 |
| Night closed leaf | NCL1 | 26,376,522 | 7,912,956,600 | 150;150 | 91.91;89.46 |
|  | NCL2 | 24,320,400 | 7,296,120,000 | 150;150 | 92.51;90.69 |
|  | NCL3 | 30,315,825 | 9,094,747,500 | 150;150 | 92.96;91.04 |
| Tough closed leaf | TCL1 | 24,615,853 | 7,384,755,900 | 150;150 | 93.19;91.75 |
|  | TCL2 | 25,771,579 | 7,731,473,700 | 150;150 | 92.77;90.35 |
|  | TCL3 | 25,735,768 | 7,720,730,400 | 150;150 | 93.05;90.73 |

**Supplementary Table 2**8 AQP genes identified and classification of the *M. bimucronata* genome

| **Species** | **Gene ID** | **Gene Name** | **Subgroup** | **Subfamily** | **Number** |
| --- | --- | --- | --- | --- | --- |
| *Mimosa bimucronata(33)* | *Mb12g014040* | MbPIP1;1 | PIP1 | PIP | 9 |
|  | *Mb07g013530* | MbPIP1;2 |  |  |  |
|  | *Mb11g001810* | MbPIP1;3 |  |  |  |
|  | *Mb04g001910* | MbPIP1;4 |  |  |  |
|  | *Mb03g002840* | MbPIP2;1 | PIP2 |  |  |
|  | *Mb06g002530* | MbPIP2;2 |  |  |  |
|  | *Mb01g001620* | MbPIP2;3 |  |  |  |
|  | *Mb03g020760* | MbPIP2;4 |  |  |  |
|  | *Mb01g016320* | MbPIP2;5 |  |  |  |
|  | *Mb03g021290* | MbTIP1;1 | TIP1 | TIP | 11 |
|  | *Mb01g001130* | MbTIP1;2 |  |  |  |
|  | *Mb01g023330* | MbTIP1;3 |  |  |  |
|  | *Mb06g001670* | MbTIP1;4 |  |  |  |
|  | *Mb11g013010* | MbTIP2;1 | TIP2 |  |  |
|  | *Mb04g015870* | MbTIP2;2 |  |  |  |
|  | *Mb10g004360* | MbTIP2;3 |  |  |  |
|  | *Mb12g015650* | MbTIP2;4 |  |  |  |
|  | *Mb03g011990* | MbTIP3;1 | TIP3 |  |  |
|  | *Mb07g020840* | MbTIP4;1 | TIP4 |  |  |
|  | *Mb01g013170* | MbTIP5;1 | TIP5 |  |  |
|  | *Mb02g015330* | MbNIP1;1 | NIP1 | NIP | 9 |
|  | *Mb02g015340* | MbNIP1;2 |  |  |  |
|  | *Mb02g011200* | MbNIP1;3 |  |  |  |
|  | *Mb05g013580* | MbNIP2;1 | NIP2 |  |  |
|  | *Mb07g015040* | MbNIP3;1 | NIP3 |  |  |
|  | *Mb12g000760* | MbNIP4;1 | NIP4 |  |  |
|  | *Mb01g033570* | MbNIP5;1 | NIP5 |  |  |
|  | *Mb04g012790* | MbNIP6;1 | NIP6 |  |  |
|  | *Mb11g011270* | MbNIP6;2 |  |  |  |
|  | *Mb02g023650* | MbXIP1;1 | XIP1 | XIP | 1 |
|  | *Mb06g004940* | MbSIP1;1 | SIP1 | SIP | 3 |
|  | *Mb05g017310* | MbSIP2;1 | SIP2 |  |  |
|  | *Mb03g025610* | MbSIP2;2 |  |  |  |

**Supplementary Table 29** Hub genes in the coexpression network in TCL

| **Name** | **Arabidosis homolog** | **Type** | **Gene symbol** | **Description** |
| --- | --- | --- | --- | --- |
| *Mb13g000970* | *AT1G18210.2* | pathwaygene | *MbCML27* | calcium-binding protein CML27 |
| *Mb11g016510* | *AT1G01260.3* | TF | *MbbHLH13* | Transcription factor |
| *Mb02g023950* | *AT2G17840.1* | pathwaygene | *MbERD7* | protein EARLY-RESPONSIVE TO DEHYDRATION 7 |
| *Mb10g014730* | *AT1G32640.1* | TF | *MbMYC2* | transcription factor |
| *Mb08g006310* | *AT5G20900.1* | TF | *MbTIFY3B* | TIFY 3B-like |
| *Mb03g029460* | *AT3G28210.1* | TF | *MbSAP* | Zinc finger AN1 domain-containing stress-associated protein |
| *Mb13g001690* | *AT5G65280.1* | pathwaygene | *MbGCL1* | lanC-like protein GCL1 |
| *Mb11g003740* | *AT3G08710.2* | pathwaygene | *MbTRXh* | Belongs to the thioredoxin family |
| *Mb06g009710* | *AT1G53210.1* | pathwaygene | *MbCaCA* | Belongs to the Ca(2 ) cation antiporter (CaCA) (TC 2.A.19) family |
| *Mb09g018000* | *AT5G21960.1* | TF | *MbERF017* | ethylene-responsive transcription factor |
| *Mb10g013830* | *AT1G10740.4* | pathwaygene | *MbPPT-1* | palmitoyl-(protein) hydrolase activity |
| *Mb10g018660* | */* | pathwaygene | *Mb10g018660* | / |
| *Mb06g012700* | *AT1G22810.1* | TF | *MbERF019* | ethylene-responsive transcription factor |
| *Mb09g024060* | *AT4G35580.1* | TF | *MbNAC* | NAC domain-containing protein |
| *Mb04g012570* | *AT5G17680.1* | pathwaygene | *Mb04g012570* | resistance protein |
| *Mb12g004010* | */* | pathwaygene | *Mb12g004010* | / |
| *Mb07g021190* | *AT4G08170.2* | pathwaygene | *MbITPK3* | Kinase that can phosphorylate various inositol polyphosphate such as Ins(3,4,5,6)P4 or Ins(1,3,4)P3 |
| *Mb13g000520* | *AT3G06500.1* | pathwaygene | *MbNINV* | Alkaline neutral invertase |
| *Mb12g016410* | *AT5G47040.1* | pathwaygene | *MbLON2* | ATP-dependent serine protease that mediates the selective degradation of misfolded and unassembled polypeptides in the peroxisomal matrix. Necessary for type 2 peroxisome targeting signal (PTS2)-containing protein processing and facilitates peroxisome matrix protein import |
| *Mb05g001870* | *AT2G28380.1* | pathwaygene | *MbDRB2* | Encodes a cytoplasmic dsRNA-binding protein DRB2 |
| *Mb01g018250* | *AT2G03500.1* | TF | *MbHHO5* | MYB family transcription factor |
| *Mb05g021560* | *AT2G37110.1* | pathwaygene | *MbCNR8* | Cell number regulator |
| *Mb03g016560* | *AT1G59650.1* | pathwaygene | *MbCW14* | Protein of unknown function (DUF1336) |
